# The role of transglutaminase 2 in stabilizing fibrin clots and supporting hemostasis

**DOI:** 10.1371/journal.pone.0354080

**Published:** 2026-08-05

**Authors:** Paulina Stoklosa, Risa Suzuki, Murielle Golomingi, Bojun Li, Manfred Heller, Kiyotaka Hitomi, Elaissa Trybus Hardy, Wilbur A. Lam, Verena Schroeder

**Affiliations:** 1 Experimental Haemostasis Research Group, Department for BioMedical Research (DBMR), University of Bern, Bern, Switzerland; 2 Laboratory of Cellular Biochemistry, Department of Basic Medicinal Sciences, Graduate School of Pharmaceutical Sciences, Nagoya University, Nagoya, Japan; 3 Proteomics & Mass Spectrometry Core Facility, Department for BioMedical Research (DBMR), University of Bern, Bern, Switzerland; 4 Wallace H. Coulter Department of Biomedical Engineering, Georgia Institute of Technology, Atlanta, United States of America; 5 Aflac Cancer and Blood Disorders Center of Children’s Healthcare of Atlanta, United States of America; 6 Department of Pediatrics, Emory University, Atlanta, United States of America; University of Bonn, Institute of Experimental Hematology and Transfusion Medicine, GERMANY

## Abstract

Transglutaminases are enzymes with pleiotropic functions in the human body, among them are the A-subunit of coagulation factor XIII (FXIII; FXIII-A) and tissue transglutaminase (TG2). FXIII has a crucial function in clot formation and stabilization. FXIII also contributes to extracellular matrix formation, wound healing and tissue regeneration, a function it shares with TG2. Whether TG2 may also have a role in hemostasis has never been studied in detail. The aim of our project was to study the role of TG2 in clot formation, stabilization and hemostasis. Here, with the use of a microfluidic endothelialized whole blood bleeding model, we detected TG2 and its crosslinking activity at the site of vessel injury, and the addition of exogenous TG2 resulted in its co-localization with the fibrin clot. In turbidimetric clot formation and lysis tests, TG2 delayed clot lysis. Finally, mass-spectrometry and proteomic analyses revealed differences in fibrin chain crosslinking patterns and protein composition of purified and plasma fibrin clots generated in the presence of either FXIII or TG2. Our data suggest that TG2 is present at the site of clot formation upon vessel injury and contributes to fibrin crosslinking and clot stabilization. Hence, TG2 may be able support hemostasis.

## Introduction

Transglutaminases are enzymes with pleiotropic functions in the human body. Prominent members of the transglutaminase family are the A-subunit of coagulation factor XIII (FXIII; FXIII-A) and tissue transglutaminase or transglutaminase 2 (TG2) [1].

FXIII plays a crucial role in hemostasis [2,3]. It circulates in plasma as a heterotetramer (FXIII-A_2_B_2_), composed of two transglutaminase A-subunits (FXIII-A) and two carrier B-subunits (FXIII-B) [4]. As the last enzyme of the coagulation cascade, FXIII-A is cleaved by thrombin, which in the presence of Ca^2+^ results in activated factor XIIIa (FXIIIa). In a transglutaminase reaction, it stabilizes the fibrin clot and makes it stiffer by introducing γ-glutamyl-ε-lysyl isopeptide bonds between fibrin γ- and α-chains [5]. Moreover, FXIII reduces the clot’s susceptibility to lysis by crosslinking antifibrinolytic proteins to the fibrin clot [6,7]. In addition to its role in coagulation, FXIII has other functions, such as stabilization of the extracellular matrix and promotion of cell migration and proliferation which is important in wound healing [8,9]. Patients with FXIII deficiency suffer from an increased bleeding tendency with a high risk of intracranial hemorrhage [10,11]. Congenital FXIII deficiency, caused by genetic mutations generally leading to the absence of circulating FXIII protein, can be treated with prophylactic FXIII replacement therapy. Acquired autoimmune FXIII deficiency, caused by autoantibodies targeting FXIII, is difficult to manage as any administered FXIII is rapidly neutralized by the autoantibodies [12,13]. Current therapeutic options include eradication of the autoantibodies with immunosuppressive therapy, however, it is burdensome for the patient, often not effective and the outcome is often poor or even fatal [14]. Therefore, there is an urgent need to improve the management of this severe bleeding disorder and novel treatment options must be explored, especially given that an increase in cases of acquired autoimmune FXIII deficiency – as for other autoimmune diseases – has been observed [15,16]. This was for us the rationale to take a closer look at TG2.

TG2 is the most ubiquitously expressed member of the TG family, it is widely distributed throughout the human body, both in extracellular spaces and in intracellular compartments of nearly all tissues [17]. In addition to its transglutaminase activity, TG2 can catalyze other types of reactions including deamidation, GTP binding/hydrolyzing, and isopeptidase activities [18]. Given the broad spectrum of its different substrates and activities, TG2 shows a great pleiotropy being involved in the regulation of numerous cellular and extracellular functions, including cell adhesion, ECM stabilization, wound healing, receptor signaling, cellular proliferation and migration [18–21]. Interestingly, TG2 has been shown to crosslink fibrinogen [22–24], and addition of TG2 to whole blood made clots more resistant to fibrinolysis [25]. Under normal conditions, TG2 is not circulating in blood. Yet, small amounts may be released from blood cells during clot formation or from endothelial cells during vessel injury and support clot formation. Furthermore, the question arises if exogenous TG2 could replace FXIII in case FXIII is absent or not functional. This would be of particular interest in the context of acquired autoimmune FXIII deficiency, as TG2 might be a candidate for a novel therapeutic agent in the treatment of this type of FXIII deficiency.

Here, with the use of a microfluidic endothelialized whole blood bleeding model [26], we detected TG2 and its crosslinking activity at the site of vessel injury, and the addition of exogenous TG2 resulted in its colocalization with the fibrin clot. In turbidimetric clot formation and lysis tests, TG2 delayed clot lysis. Finally, we used mass-spectrometry and proteomic analyses to examine fibrin chain crosslinking and protein composition of purified and plasma fibrin clots generated in the presence of either FXIII or TG2. We also performed preliminary experiments in the presence of FXIII inhibitors.

## Materials and Methods

### Microfluidic bleeding model experiments

The microfluidic bleeding model was developed and characterized in detail as described by Sakurai et al. [26]. The devices were fabricated from polydimethylsiloxane and endothelial cells were seeded into the model as described earlier [27.28]. The dimensions of the vascular channels (150x50 µm, which are reduced and smoothend by coating and growth of the endothelial cell monolayer) make the model comparable to human arterioles and venules. The devices were coated with a collagen solution (Rat tail collagen I; Thermo Fisher Scientific), followed by a fibronectin solution (0.005% in DPBS; Merck). Confluent HUVEC cells (Lonza), passage 5, were trypsinized and centrifuged. The cell pellet was resuspended in a dextran solution (80 µg/mL in cell culture medium) and introduced into the main channel of each device. The devices were incubated for one hour, then connected to a syringe containing cell culture medium and perfused for 48 hours at an initial flow rate of 1 µL/min for the first 12 hours, followed by 2 µL/min for the remaining 36 hours, allowing the cells to form a confluent endothelial monolayer.

The bleeding experiments were carried out as previously described [27,28]. We used fresh citrated whole blood from anonymous healthy donors obtained from the blood donation center in Bern, Switzerland, between 19^th^ September 2019 and 13^th^ December 2024. The donors gave global consent at the blood donation center that their samples may be used for research purposes. Because we obtained the samples completely anonymous, no approval of the ethic’s committee was required for this study.

The obtained whole blood was supplemented with 40 µg/mL of corn trypsin inhibitor (CTI; Loxo) to prevent contact activation of coagulation in the syringe and tubing. As described by Sakurai et al. [26], the trigger of blood coagulation is the mechanical injury causing disrupture of the endothelial cell monolayer – leading to release of von Willebrand factor – and exposure of the subendothelial coating of collagen – leading to platelet adhesion and activation and formation of a platelet plug (primary hemostasis) which also activates the coagulation cascade leading to fibrin formation (secondary hemostasis). The endothelial cells grown in the device were stained with Cellmask™ Orange plasma membrane stain (1 µL/mL; Thermo Fisher Scientific). The device was then placed on the stage of a Zeiss LSM 710 confocal microscope equipped with Airyscan. A 2 mL vacuum was applied to the valve channel. Just before the start of the measurement, the blood sample was recalcified to a final concentration of 12.5 mM Ca^2+^ and perfused through the main channel at a flow rate of 2 µL/min. As described by Sakurai et al. [26] a flow rate of 2 μL/min in this model corresponds to a shear rate of 500 s^-1^ which is comparable to a venous shear rate. A pressure was applied via the side channel to induce injury to the main channel, and images of the injury site were captured.

Clot formation was detected and quantified by measuring fibrin formation over time. To detect fibrin formation, the blood samples were supplemented with a fibrinogen-Alexa Fluor™ 488 or 647 conjugate (10 µL/500 µL of blood; Invitrogen, Thermo Fisher Scientific). Transglutaminase activity was detected by incorporation of fluorescently labelled (FITC or TAMRA) peptide substrates for FXIII (pepF11; n = 6 experiments) and/or TG2 (pepT26; n = 6 experiments) used at 1µM final concentration [29,30]. To detect endogenous TG2 protein (n = 5 experiments), we used two primary antibodies, one to the catalytic domain (clone XTG17, A033, Zedira) and one to the beta sheet domain (clone XTG11, A034, Zedira). For TG2-fibrinogen colocalization studies (n = 5 experiments), recombinant TG2 (T022, Zedira) was added to the blood samples at the final concentration of 50 µg/mL. For the experiments with FXIII inhibitor, ZED1301 (Zedira) was used at the final concentration of 250 nM. ZED1301 is a site specific irreversible inhibitor of FXIII that shows 30-fold selectivity for FXIII compared to TG2 (IC50 for FXIII = 100 nM, IC50 for TG2 = 3000 nM) [31]. A monoclonal antibody to detect crosslinked fibrin (DD-XLink-mab, A076, Zedira) was used at the concentration of 2 µL/500 µL of blood. To detect primary antibodies a goat anti-mouse Alexa Fluor™ 647-labelled secondary antibody was used (A-21236, Invitrogen, Thermo Fisher Scientific).

The relative fluorescence signal intensities were measured over time in a region of interest (ROI) of 300x300 µm^2^ around the injury site. The fluorescence intensity was measured with ImageJ (Fiji) [32] and corrected against background fluorescence intensity.

### Turbidimetric clot formation and lysis assay

Effects of TG2 and FXIII on plasma clot formation and lysis were investigated by measuring changes in absorbance over time. To wells of a low-binding 96-well plate (Greiner Cellstar^®^, Merck) 15 µL activation mix followed by 85 µL of diluted plasma (final plasma concentration 50%) were added and the measurements were started. Plates were shaken (510 rpm, 10 seconds) and read at 340 nm every 12 seconds for up to 2 hours in a Tecan Reader Spark 10M microplate reader. All measurements were performed in duplicate. The activation mix contained final concentrations of tissue factor (Dade^®^ Innovin^®^, Siemens Healthineers) diluted 1:1250, 17mM CaCl_2_, 200 ng/mL rtPA (Actilyse, Boehringer Ingelheim), 10 µM phospholipids (Phospholipid-TGT, Rossix) in assay buffer (25 mM HEPES, 137 mM NaCl, 3.5 mM KCl, and 1% BSA, pH 7.4). Data analysis was performed using the Shiny App tool (https://drclongstaff.shinyapps.io/clotlysisCL_2019) [33] to determine parameters such as time to 50% clotting time, 50% clot lysis time, maximum absorbance, and time from 50% clotting to 50% clot lysis (the latter being more robust when maximum clot absorbance presents as a broad plateau). We used 50% lysis when investigating clot lysis because 100% lysis time and absorbance is often difficult to pin down precisely. For each individual measurement, the absorbance at the starting point was set as baseline and absorbance values over time were expressed relative to that baseline. Where we wanted to directly compare curves visually, we normalized them by setting the absorbance to 1 for the first time point of all curves, but this does not affect times to clotting or lysis.

We used either normal reference plasma (CRYOcheck™ Normal Reference Plasma, Precision BioLogic; all coagulation factor levels including FXIII and fibrinogen were in the normal range as confirmed in the certificate of analysis, with fibrinogen 2.83 g/L, and FXIII 1 U/mL), FXIII-depleted plasma (Milan Analytica; FXIII levels were <0.01 U/mL while other coagulation proteins and coagulation tests (PT, aPTT) were in the normal range), or plasma obtained from a patient with autoimmune FXIII deficiency we described earlier [14]. In the patient sample used in this experiment, no FXIII activity and no FXIII antigen were detectable and the amount of inhibitor was determined as 20 Bethesda Units. We added either recombinant FXIII-A_2_ (Zedira; final concentration 70 nM) or recombinant TG2 (Zedira; final concentration 140 nM). In other experiments we added the FXIII inhibitor ZED1301 (Zedira; final concentration 250 nM), or a purified anti-FXIII autoantibody with an IC50 value of 170 µg/mL isolated from a patient with autoimmune FXIII deficiency [34] (final concentration 400 µg/mL; a kind gift from Z. Bagoly, E. Katona and L. Muszbek, University of Debrecen, Hungary) which we incubated with normal reference plasma for 1 hour prior to the experiment.

### Generation of fibrin and plasma clots for mass spectrometry

Fibrin clots were generated from FXIII-free peak 1 fibrinogen (10 mg/mL, Enzyme Research Laboratories), recombinant FXIII-A_2_ (70 nM, Zedira) or recombinant TG2 (140 nM, Zedira), thrombin (2 U/mL, from human plasma, 605190, Sigma Aldrich), and CaCl₂ (17 mM), all final concentrations in assay buffer 1 (0.05 mol/L Tris-HCl, 0.1 mol/L NaCl, pH 7.4). The mixture was incubated at 37°C for 30 minutes with gentle shaking (350 rpm) to allow the formation of the fibrin clot. In total, we analyzed two purified clots crosslinked with FXIII and two clots crosslinked with TG2.

Plasma clots were prepared from normal reference plasma (CRYOcheck™ Normal Reference Plasma) (control clots), or FXIII-depleted plasma (Milan Analytica) supplemented with either recombinant FXIII-A_2_ (Zedira) or recombinant TG2 (Zedira). For each sample, 200 µL of plasma was mixed with 140 µl assay buffer 2 (25 mM HEPES, 137 mM NaCl, 3.5 mM KCl, and 1% BSA, pH 7.4) containing either FXIII-A_2_ (final concentration 70 nM) or TG2 (final concentration 140 nM), and clotting was induced with 43 µL activation mix (Innovin, final dilution 1:70; CaCl_2_, final concentration 17 mM). The samples were incubated at 37°C for 30 minutes with gentle shaking (350 rpm). In total, we analyzed seven clots made from normal plasma, seven clots made from FXIII-depleted plasma with recombinant FXIII added, and seven clots made from FXIII-depleted plasma with recombinant TG2 added.

Following clot formation, the samples were subjected to a series of washing and buffer exchange steps at room temperature. First, the clot was centrifuged at 9,000 x g for 3 minutes to remove any residual fluid. The supernatant was discarded, and the clot was shaken with 500 µL of the respective assay buffer (1 or 2) for 20 minutes, followed by a second centrifugation at 9,000 x g for 3 minutes. This process was repeated with 500 µL of 6M guanidine hydrochloride solution for 30 minutes, followed by a final centrifugation at 9,000 x g for 3 minutes.

In case of plasma clots, the clot was additionally shaken with 500 µL of 6M guanidine hydrochloride solution at 4°C for 16 hours (overnight). After the overnight incubation, the buffer was changed to fresh assay buffer, and the clot was vortexed again before the final centrifugation step at 9,000 x g for 3 minutes at room temperature.

Following these steps, the clots were stored at −20°C until processed for mass spectrometry analysis.

### Mass spectrometry and proteomic data analysis

The detailed procedures are described in the supporting information. Briefly, the clots were solubilized, proteins were coupled to magnetic beads and digested. Digests were analyzed by LC-MS/MS on a system consisting of a Vanquish Neo UPLC coupled with an Orbitrap Astral mass spectrometer (ThermoFisher Scientific, Bremen, Germany). The mass spectrometry data were processed with FragPipe software (v.22.0). The list of identified proteins was filtered to proteins having at least 4 razor peptides identified and having known cross-linker annotation and/or being a known interactor with fibrinogen. This reduced protein sequence database was then used for the identification of K-Q cross-linked peptides with ProteomeDiscoverer 3.1 and its XlinkX plugin.

## Results

We performed experiments using purified proteins, plasma and a whole blood endothelialized microfluidic bleeding model to investigate the effects of TG2 on fibrin crosslinking and clot formation and stabilization, and to elucidate whether TG2 may support clot formation and stabilization and hence hemostasis in the absence of FXIII and even in the presence of FXIII inhibitors.

### TG2 is present and functional at the injury site in a microfluidic bleeding model

**Fig 1:**
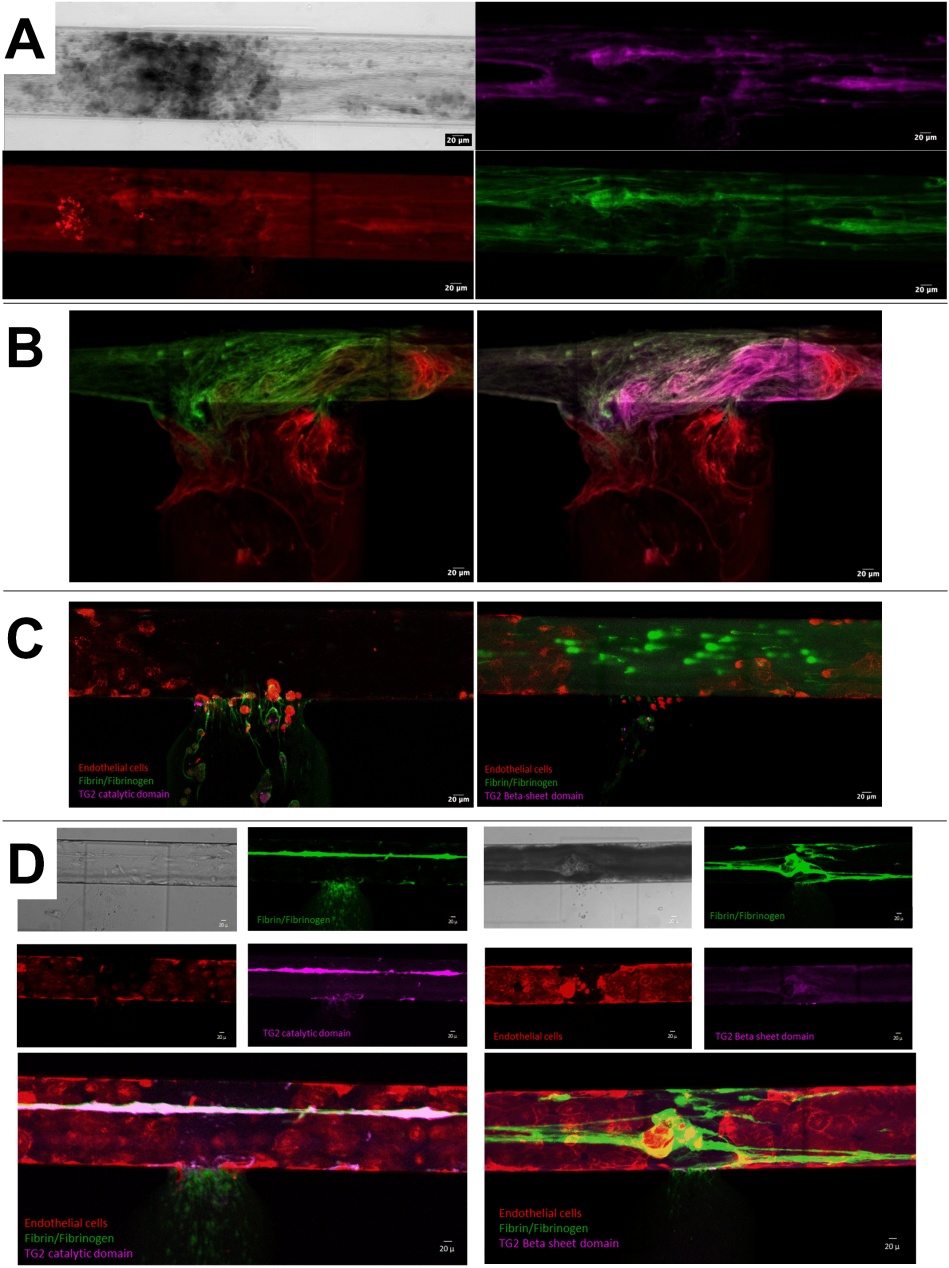
TG2 transglutaminase activity and protein are present at the injury site in a microfluidic bleeding model. **(A)** In these images taken 60 minutes after inducing the injury, deposition of fibrin (purple) and *in situ* TG2 activity by incorporation of TAMRA-labelled (red) pepT26 (final concentration of 1 µM) and *in situ* FXIII activity by incorporation of FITC-labelled (green) pepF11 (final concentration of 1 µM) were detected at the injury site. **(B)** In these images taken 70 minutes after inducing the injury, FXIII *in situ* activity (green) and TG2 *in situ* activity (red) showed some overlap with each other and fibrin deposition (purple), highlighted by yellow and white co-localization in the right-hand panel, but also distinct localization. Panels A and B represent experiments performed with blood from 6 different donors (n = 6). **(C)** Endogenous TG2 protein was detected with antibodies that either targeted the beta-sheet domain or core domain of TG2 (purple). Endothelial cells were stained with CellMask (red) and fibrin deposition was detected by addition of fluorescently labelled fibrinogen (green). **(D)** Addition of exogenous recombinant TG2 (50 µg/mL) resulted in strong signals for TG2 (purple) with both antibodies (left-hand side: beta-sheet domain TG2 antibody; right-hand side: core domain TG2 antibody) at the injury site and co-localization with fibrin (green). Panels C and D represent experiments performed with blood from 5 different donors (n = 5).

To examine whether endogenous TG2 transglutaminase activity can be detected at the injury site, fluorescently-labelled peptide substrates specific for either TG2 or FXIII-A were added to recalcified whole blood from healthy blood donors. As shown in Fig 1A, the specific peptide substrate for FXIII-A, FITC-labelled F11 (FITC-pepF11, in green), co-localized with fibrin (in purple). A control peptide with abolished crosslinking site (FITC-F11QN) was not incorporated into the clot (shown in supporting information). The specific peptide substrate for TG2, TAMRA-labelled T26 (TAMRA-pepT26, in red) could also be detected in the clot. As shown in Fig 1A, the TG2 substrate (red) showed some co-localization with fibrin and FXIII substrate (green), but interestingly also some distinct localization (Fig 1B). When a TG2 inhibitor (Z006) was used, significantly reduced incorporation of TAMRA-T26 was observed (shown in supporting information).

Next, we used antibodies against the TG2 catalytic domain and the TG2 beta sheet domain to visualize endogenous TG2 protein. We were able to detect small amounts of TG2 (purple signal) at the injury site with both antibodies (Fig 1C), however, we observed a substantial variability between experiments. Over all the experiments (n = 5), TG2 was detectable five times (three times with the beta sheet domain antibody and twice with the core domain antibody), and it was not detectable three times (once with the beta sheet antibody and twice with the core domain antibody). Wondering whether TG2 might be released from activated platelets, we performed an additional experiment using flow cytometry (which is described in the supporting information), but we did not find any evidence for a release of TG2 from activated platelets. When we added exogenous recombinant TG2 (50 µg/mL) to the blood, we observed strong co-localization of TG2 signals (purple), obtained with both TG2 antibodies, with fibrin (green) formed at the injury site (Fig 1D).

### TG2 crosslinks fibrin in the presence of a FXIII inhibitor in the microfluidic bleeding model

**Fig 2:**
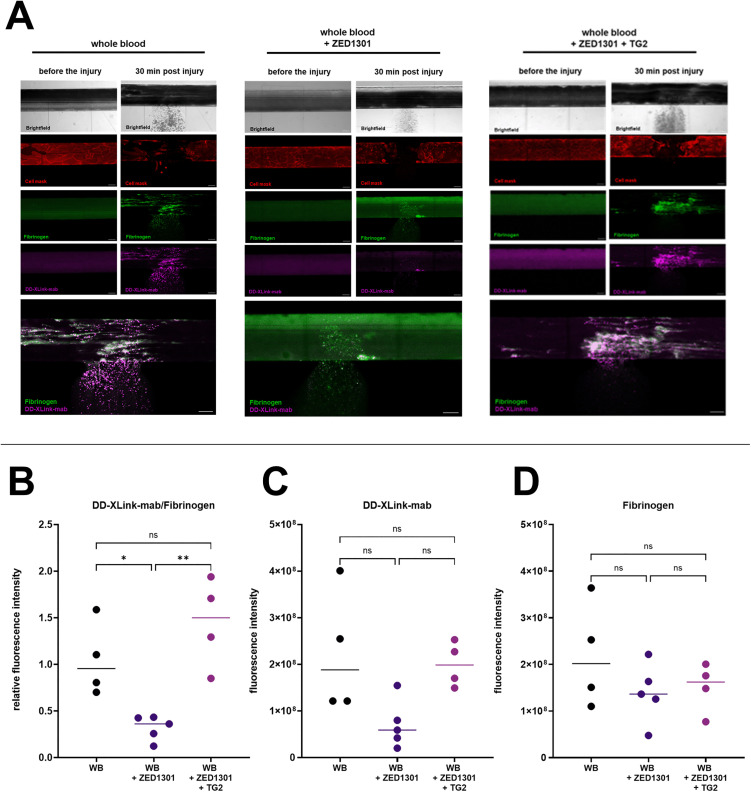
TG2 crosslinks fibrin in the presence of a FXIII inhibitor. **(A)** Endothelial cells were stained with CellMask (red), whole blood was supplemented with fluorescently-labelled fibrinogen (green) and DD-XLink-mab antibody to visualize fibrin crosslinks (purple). Whole blood was either untreated (control; n = 4), treated with 250 nM FXIII inhibitor ZED1301 (n = 5), or treated with 250 nM FXIII inhibitor ZED1301 and 140 nM recombinant TG2 (n = 4). Before the injury, the blood was flowing from left to right. When the injury was induced, blood flowed out towards the side channel and fibrin formation (green) and fibrin crosslinking (purple) were observed. Fluorescence intensity was measured in the area of the injury (300 µm x 300 µm). **(B)** Ratio of fibrin crosslinks (DD-XLink-mab) relative to the total fibrin signal. **(C)** Fibrin crosslinks (DD-XLink-mab) fluorescence signal intensity. **(D)** Fibrin fluorescence signal intensity. Data were analyzed with ImageJ and statistical analysis was performed with GraphPad Prism (One-way ANOVA, *P < 0.05, **P < 0.005, ***P < 0.0005).

As shown in Fig 2A, we performed bleeding model experiments in the presence of the FXIII inhibitor ZED1301 and measured the intensity of the fibrin signal (green) and the signal for crosslinked fibrin using the DD-XLink-mab (purple). The FXIII inhibitor ZED1301 reduced the ratio of crosslinked fibrin/total fibrin significantly (Fig 2B), and this was due to significantly reduced fibrin crosslinking (Fig 2C), while the signal for fibrin deposition was not affected by FXIII inhibition (Fig 2D). Addition of TG2 was able to restore fibrin crosslinks even in the presence of the FXIII inhibitor (Figs 2B,C).

### TG2 prolongs clot lysis

Seeing direct evidence in the microfluidic bleeding model for the capability of TG2 to introduce fibrin crosslinks, we aimed to examine the effects of TG2 on fibrin clot formation and lysis more closely using a turbidimetric clot formation and lysis assay.

**Fig 3:**
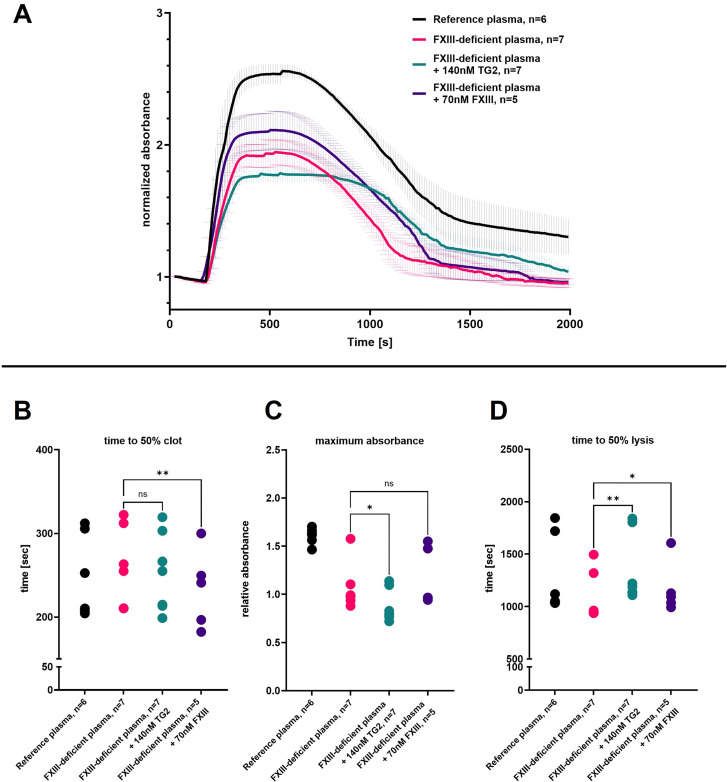
Turbidimetric clot formation and lysis assay of FXIII-depleted plasma supplemented with TG2 or FXIII-A_2_. Experiments were performed in 5-7 replicates with the same batches of plasma (n = 5-7) as indicated in the panels. **(A)** Absorbance curves over time in normal reference plasma and FXIII-depleted plasma, and with addition of 70 nM FXIII-A_2_ or 140 nM TG2. Absorbance values were normalized to 1 for the first time point (in order to be able to directly compare visually the mean traces of the 4 different samples) and are displayed as mean and SEM. **(B)** Clot formation time was represented as average time from beginning of clot formation to 50% of the maximum absorbance. **(C)** Average maximum absorbance (shown as relative absorbance values in regard to the individual starting point of the measurement set as baseline for each individual curve). **(D)** Clot lysis time was expressed as time from 50% clotting to 50% clot lysis. Data were analyzed with the Shiny app and statistical analysis was performed with GraphPad Prism (mixed-effects model, *P < 0.05, **P < 0.005, ***P < 0.0005).

When we performed turbidimetric clot formation and lysis assays in FXIII-depleted plasma supplemented with TG2 or FXIII-A_2_ (Fig 3A), we found that FXIII-A_2_ but not TG2 reduced the time to 50% clot formation (Fig 3B). Interestingly, TG2 but not FXIII-A_2_ reduced the maximum absorbance (Fig 3C). Both TG2 and FXIII-A_2_ significantly prolonged clot lysis, expressed as time from 50% clotting to 50% lysis (Fig 3D).

**Fig 4:**
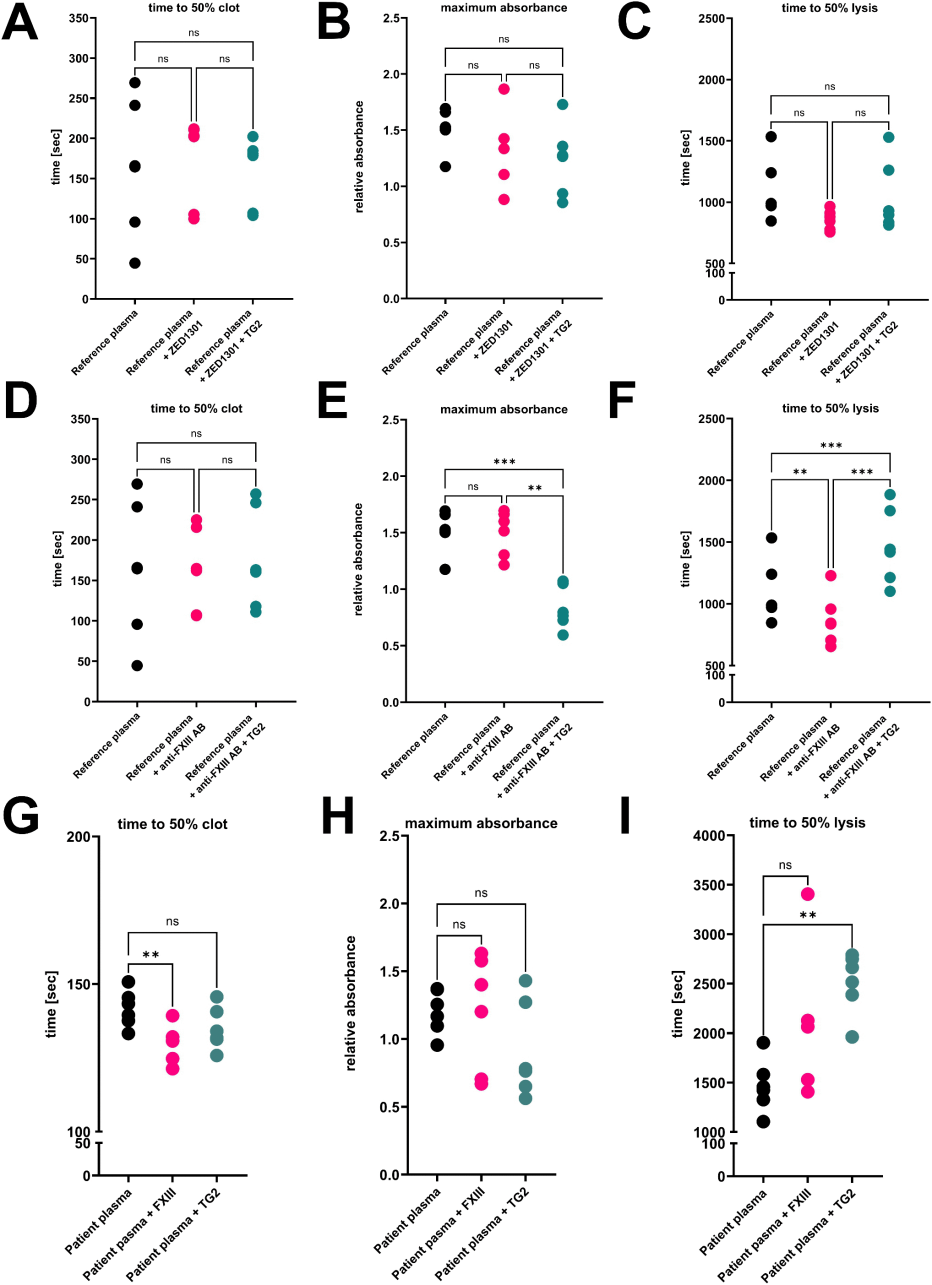
Turbidimetric clot formation and lysis assay and effects of FXIII-A_2_ and TG2 in the presence of FXIII inhibitors. Experiments were performed in 5 replicates with the same batches of plasma (n = 5). **(A-C)** Turbidimetric clot formation and lysis assays were performed in normal reference plasma, with addition of the FXIII inhibitor ZED1301 (250 nM), and recombinant TG2 (140 nM). **(A)** Clot formation time was represented as average time from beginning of clot formation to 50% of the maximum absorbance. **(B)** Average maximum absorbance. **(C)** Clot lysis time was expressed as time from 50% clotting to 50% clot lysis. **(D-F)** Turbidimetric clot formation and lysis assays were performed in normal reference plasma, incubated with an autoantibody against FXIII purified from a patient with acquired autoimmune FXIII deficiency (400 µg/mL), and recombinant TG2 (140 nM). **(G-I)** Turbidimetric clot formation and lysis assays were performed in plasma from a different patient with acquired autoimmune FXIII deficiency supplemented with recombinant FXIII-A_2_ (70 nM) or recombinant TG2 (140 nM). Data were analyzed with the Shiny app and statistical analysis was performed with GraphPad Prism (mixed-effects model, *P < 0.05, **P < 0.005, ***P < 0.0005).

Next, we tested the effects of two types of FXIII inhibitors in normal reference plasma, the commercial inhibitor ZED1301 and an autoantibody isolated from the blood of a patient with autoimmune FXIII deficiency, and we tested the performance of TG2 in the presence of both inhibitors (Fig 4). No statistically significant effects on clot formation time, maximum absorbance or clot lysis were obtained with ZED1301 (Figs 4 A-C), but we observed a trend towards faster clot lysis with ZED1301 and prolongation of clot lysis when TG2 was added on top (Fig 4C). Addition of the FXIII autoantibody had no effects on clot formation time (Fig 4D), but significantly reduced clot lysis time, and addition of TG2 reversed that effect and prolonged clot lysis significantly (Fig 4F). Finally, we used plasma from another patient with autoimmune FXIII deficiency and supplemented it with either FXIII-A_2_ or TG2. The turbidity curve obtained with this patient’s plasma shows that a clot is formed but it is quickly dissolved (supporting information). Addition of FXIII-A_2_ reduced time to 50% clot formation (Fig 4G), but was not able to stabilize the clot and prolong clot lysis to the same extent as TG2 did (Fig 4I).

### TG2 shows distinct fibrin chain crosslinking patterns and protein incorporation into plasma clots

Seeing some differences in plasma clot stabilization between FXIII and TG2, we aimed to investigate this more closely by analyzing the crosslinking pattern of fibrin chains in clots made from purified proteins, and by comparing the protein composition of plasma clots.

**Fig 5:**
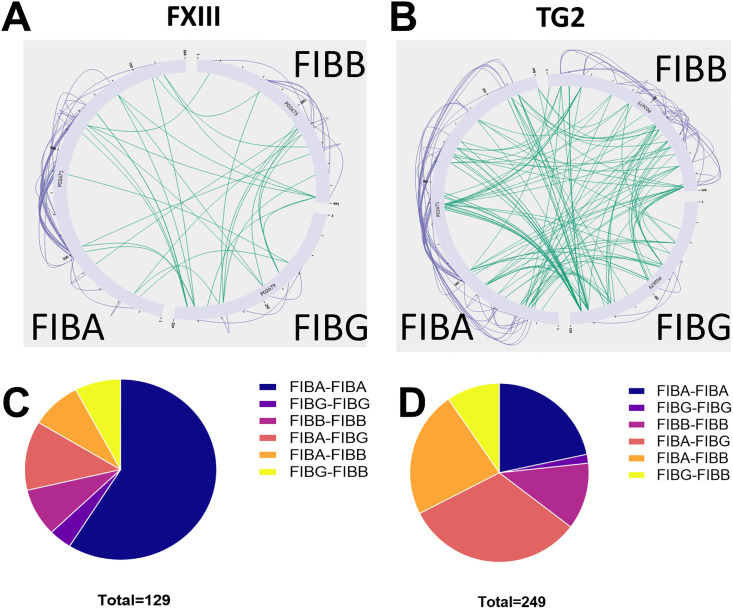
FXIII-A_2_ and TG2 crosslinking patterns of fibrin chains. Fibrin clots were generated from FXIII-free fibrinogen and recombinant FXIII-A_2_ or TG2, clotted with thrombin and Ca^2+^. Crosslinks between fibrin chains introduced by **(A)** FXIII-A_2_ or **(B)** TG2. Graphs were generated with xiView software1 [35]. **(C-D)** The relative distribution of crosslinks between different fibrin chains. FIBA: fibrinogen alpha (UniProt ID P02671), FIBB: fibrinogen beta (UniProt ID P02675), FIBG: fibrinogen gamma (UniProt ID P02679).

As shown in Fig 5, purified fibrin clots generated with FXIII-A_2_ were characterized by a strong preference for crosslinks between fibrin alpha-alpha chains, followed by crosslinks between fibrin alpha-gamma chains (Figs 5A,C). Interestingly, we also observed some crosslinking sites on fibrin beta chains. The TG2 crosslinking pattern showed higher diversity, involving a higher number of different crosslinking sites on all three fibrin chains (Figs 5B,D). When we analyzed the protein composition of plasma clots made from the same FXIII-depleted plasma in the presence of either FXIII-A_2_ or TG2 (Table 1), we could confirm that FXIII-A_2_ was only present in the FXIII-clots and TG2 was only present in the TG2-clots. FXIII showed a clear preference to incorporate fibrin, α_2_-antiplasmin, and thrombospondin into the clots, while TG2 favoured coagulation factors V, IX and XII, protein C and protein S, plasminogen, albumin, and complement components C6 and factor H.

**Table 1:** Proteins identified in plasma clots formed with FXIII-A_2_ or TG2. The table contains plasma proteins relevant to clot formation and lysis, or known targets of either FXIII-A_2_ or TG2, that were detected in clots generated in FXIII-depleted plasma with the addition of either FXIII-A_2_ or TG2. The heat coloring represents the distribution of the identified proteins between the samples in terms of the number of detected spectral matches, e.g., of the total number of spectral matches for albumin, 31% were detected in clots formed with FXIII-A_2_ and 69% were detected in clots formed with TG2.

no.	Protein	UniProt ID	% of total (FXIII)	% of total (TG2)
1	Albumin	P02768	31%	69%
2	Alpha-2 macroglobulin	P01023	43%	57%
3	Alpha-2 plasmin inhibitor	P08697	72%	28%
4	Apolipoprotein A-II	P02652	43%	57%
5	Apolipoprotein A-IV	P06727	37%	63%
6	Apolipoprotein C-I	P02654	33%	67%
7	Apolipoprotein C-II	P02655	56%	44%
8	Apolipoprotein C-III	P02656	33%	67%
9	Apolipoprotein D	P05090	37%	63%
10	Apolipoprotein E	P02649	39%	61%
11	Apolipoprotein M	O95445	45%	55%
12	Apolipoprotein(a)	P08519	44%	56%
13	Carboxypeptidase N catalytic chain	P15169	35%	65%
14	Coagulation factor IX	P00740	22%	78%
15	Coagulation factor V	P12259	23%	77%
16	Coagulation factor X	P00742	40%	60%
17	Coagulation factor XII	P00748	18%	82%
18	Coagulation factor XIII A chain	P00488	100%	0%
19	Complement C2	P06681	40%	60%
20	Complement C3	P01024	46%	54%
21	Complement C4-B	P0C0L5	47%	53%
22	Complement C8 alpha chain	P07357	37%	63%
23	Complement C5	P01031	50%	50%
24	Complement component C6	P13671	25%	75%
25	Complement component C7	P10643	32%	68%
26	Complement component C8 beta chain	P07358	45%	55%
27	Complement component C8 gamma chain	P07360	44%	56%
28	Complement component C9	P02748	44%	56%
29	Complement factor B	P00751	31%	69%
30	Complement factor D	P00746	0%	100%
31	Complement factor H	P08603	30%	70%
32	Complement factor I	P05156	35%	65%
33	Extracellular matrix protein 1	Q16610	36%	64%
34	Fibrinogen alpha chain	P02671	68%	32%
35	Fibrinogen beta chain	P02675	60%	40%
36	Fibrinogen gamma chain	P02679	60%	40%
37	Fibronectin	P02751	37%	63%
38	Fibulin-1	P23142	35%	65%
39	Fibulin-2	P98095	37%	63%
40	Galectin-3-binding protein	Q08380	41%	59%
41	Histidine rich glycoprotein	P04196	41%	59%
42	Immunoglobulin heavy constant gamma 1	P01857	34%	66%
43	Immunoglobulin heavy constant gamma 3	P01860	38%	62%
44	Plasminogen	P00747	31%	69%
45	Protein-glutamine gamma-glutamyltransferase 2 (TG2)	P21980	0%	100%
46	Prothrombin	P00734	36%	64%
47	Thrombospondin	P07996	71%	29%
48	Vitamin K-dependent protein C	P04070	23%	77%
49	Vitamin K-dependent protein S	P07225	25%	75%
50	Vitronectin	P04004	46%	54%
51	Von Willebrand factor	P04275	36%	64%

## Discussion

We set out to elucidate if TG2 could support clot formation and stabilization and contribute to hemostasis. TG2 is not a classical plasma protein and is not reported to circulate in plasma in signifcant amounts under normal circumstances, but it can occur in plasma in situations of tissue damage, e.g., TG2 has been detected in the serum from patients with chronic kidney disease [36]. We were able to detect small amounts of endogenous TG2 *in situ* activity and protein at the site of vessel injury, but the results were inconsistent. As TG2 is present in endothelial cells and blood cells including erythrocytes or macrophages [37], it is possible that small amounts of TG2 have been released into whole blood from healthy blood donors due to endothelial damage or hemolysis during blood sampling and/or plasma preparation. On the other hand, release from damaged endothelial cells upon induction of the vessel injury in our microfluidic model may be another possibility, as the injury sites can vary in size and the presence of TG2 may depend on the extent of endothelial layer damage. Finally, release of TG2 from damaged red blood cells, but not platelets, during vessel injury and hemostatic response in our microfluidic model may be another explanation. However, those hypotheses require further investigation.

Importantly, when we added recombinant TG2 to whole blood in the microfluidic bleeding model, it did co-localize with fibrin clot formation at the injury site and was capable of introducing fibrin crosslinks even in the presence of a FXIII inhibitor. As demonstrated by turbidity measurements, TG2 prolonged clot lysis. Interestingly, TG2 plasma clots showed lower turbidity which may be a result of different clot architecture or protein composition. Indeed, mass spectrometry analysis revealed differences in fibrin chain crosslinking patterns between FXIII-A and TG2 as well as differences in proteins incorporated into plasma clots. These observations are in line with the broader substrate specificity of TG2 compared with FXIII-A [29,38].

Interestingly, we found evidence for crosslinking of fibrin beta-chains in purified fibrin clots generated in the presence of TG2 but also FXIII-A. A modifying influence of TG2 on fibrin crosslinking in acute liver disease was recently also shown by Wei et al [39]. Crosslinking of fibrin beta-chains by FXIII-A was excluded by early studies [40], but Lund Nikolajsen et al [41] also reported the fibrinogen beta-chain as a substrate for FXIII-A. More detailed proteomic analyses including identification of individual crosslinking sites are planned for a follow-up project.

Eventually, we were interested to explore in some pilot experiments if administration of exogenous TG2 could support fibrin clot formation and stability and hence haemostasis, when FXIII-A is absent or non-functional because of autoantibodies in patients with autoimmune FXIII deficiency. In the turbidimetric assay, TG2 prolonged clot lysis better than FXIII-A. Futher experiments are certainly needed to confirm functioning of TG2 while FXIII activity is abolished. Whether certain autoantibodies against FXIII-A might also crossreact and inhibit/neutralize TG2 also needs further investigation.

Our study has of course limitations. Our aim was to present merely proof-of-concept that TG2 can stabilize fibrin clots and support hemostasis. Our study was motivated by open needs in the treatment of acquired autoimmune FXIII deficiency. However, before TG2 may even be considered as a potential novel treatment option, additional questions have to be addressed and futher in vitro and in vivo studies need to be performed. Since TG2 is normally not circulating in plasma, we have chosen experimental TG2 concentrations according to FXIII plasma concentrations. However, whether the chosen TG2 concentrations would be tolerated *in vivo* would need to be determined in future *in vivo* studies which must also investigate TG2 kinetics when administered intravenously, its efficacy to stop and prevent bleeding episodes and its safety considerations, including off-target crosslinking and potential prothrombotic effects. Furthermore, crosslinking of fibrin and other proteins by TG2 and implications for clot structure-function under close-to-physiological conditions must be better understood.

Taken together, with the microvascular bleeding model we could visualize for the first time the presence of TG2 at the site of vascular injury and clot formation. Our data also suggest that TG2 is indeed able to crosslink fibrin, support fibrin formation and clot stability, resulting in delayed clot lysis, even in the presence of FXIII inhibitors. We therefore believe that it would be worth investing more research into TG2 as a potential therapeutic approach for patients with autoimmune FXIII deficiency.

## Supporting information

S1 File Supporting information. (DOCX)
