# Supplementary material for: The role of transglutaminase 2 in stabilizing fibrin clots and supporting hemostasis
Source: PLoS One. 2026 Aug 5;21(8):e0354080. doi: 10.1371/journal.pone.0354080 (PMC13440824; doi:10.1371/journal.pone.0354080)
Supplement: Supplementary file 1 [file pone.0354080.s001.docx]

**Supporting information**

**Detailed methods for mass spectrometry and proteomic data analysis**

Clots were centrifuged for 20 min at 16`000x g and 4 °C to remove storage solution. Clots were covered by 50 μL hydroxylamine solution (4.5 M Gnd-HCl, 1 M NH_2_OH-HCl, 0.2 M K_2_CO_3_ pH 9.0). Samples were briefly vortexed then incubated at 45 °C with 300 rpm shaking on a Thermomixer for 16 hours. Following incubation, samples were centrifuged for 1 min at 16`000x g and 20 °C and the pre-digest supernatant was transferred into a new tube without disturbing the pellet. The protein concentration was determined by Qubit Protein Assay (ThermoFisher Scientific). An aliquot corresponding to 15 μg was diluted with 10 μL 8 M urea/50 mM Tris-HCl pH 8.0 and cysteines reduced and alkylated with 10 mM DTT and 50 mM iodoacetamide by successive incubations for 30 min at 37 °C and at room temperature, respectively. Upon quenching of excess iodoacetamide, 6 μL of SP3 bead slurry (combination of hydrophilic and hydrophobic Sera-Mag Speedbeads at 25 mg/mL, Cytiva) was added and proteins captured by addition of ethanol to a final concentration of 50 % and incubation for 15 min at room temperature. The liquid was removed, and beads were washed three times with 100 μL 80 % ethanol. To the dry beads 15 μL of 8 M urea/50 mM Tris-HCl pH 8.0 was added, the urea was diluted to 1.6 M with 20 mM Tris-HCl pH 8.0/ 2 mM CaCl_2_ and proteins digested two times for 2 hours at 37 °C by adding 300 ng sequencing grade trypsin (Promega). After digestion, the SP3 beads were captured on a magnet and the supernatant transferred into a new vial for acidification with 1/20 volume of 20 % tri-fluoro-acetic acid. An aliquot of 3 μL digest (500 ng) was analyzed by LC-MS/MS on a system consisting of a Vanquish Neo UPLC coupled with an Orbitrap Astral mass spectrometer (ThermoFisher Scientific, Bremen, Germany). Digests were loaded onto a pre-column (PepMap C18, 5 μm, 100 Å, 300 μm × 5 mm) and peptides were eluted in back flush mode onto the analytical nano-column (Waters CSH C18, 1.9 μm, 100 Å, 0.075 mm i.d. × 200 mm) using an acetonitrile gradient of 5-40 % solvent B (0.1 % FA in water/acetonitrile 4,9:95) in 22 min at a flow rate of 300 nL/min. The column effluent was directly coupled to the mass spectrometer via a nanoflex electrospray source (Thermo Fischer, Bremen, Germany). Precursor ion scans were recorded in the orbitrap detector with resolution of 240,000 (at *m*/*z* = 250) every 0.6 sec in the m/z range of 380-980, maximum injection time (IT) of 5 ms, and an automatic gain control (AGC) setting of 3’000’000. Precursor ions with charge 2-6 were fragmented with a normalized high energy collision energy of 27 % and fragment spectra were acquired parallel to the full scan in the Astral detector at a maximum IT of 5 ms, AGC of 8’000, and exclusion from further fragmentation for 20 sec.

The mass spectrometry data were processed with FragPipe software (v.22.0) against concatenation of the following databases: SwissProt Homo Sapiens (release 2025_01), and 280 proteins commonly found as contaminants as described elsewhere^3^. The list of identified proteins was filtered to proteins having at least 4 razor peptides identified and having known cross-linker annotation and/or being a known interactor with fibrinogen. This reduced protein sequence database was then used for the identification of K-Q cross-linked peptides with ProteomeDiscoverer 3.1 and its XlinkX plugin allowing C-terminal peptide cleavage after KRN if no P follows, oxidation of M and P, deamidation of N, protein N-terminal acetylation as variable modifications, and carbamidomethlyation of C as fixed modification, respectively. Cross-linked peptides were filtered to a 1 % false discovery rate.

**Control experiments in the microfluidic model**

Incorporation of the FXIII substrate peptide FITC-F11 could be seen in the upper channel, while no incorporation of the control peptide with abolished crosslinking site FITC-F11QN was seen in the lower channel.

Incorporation of TG2 substrate peptide TAMRA-T26 could be clearly seen in the upper channel, while it was significantly reduced in the presence of TG2 inhibitor Z006 in the lower channel.

**Additional experiments on potential TG2 release from platelets**

Whole blood samples were obtained from anonymous donors at the local blood donation center.

To assess if TG2 is present on activated platelets, analysis was performed according to the platelet activation protocol of BD Biosciences: 200 µM adenosine diphosphate (ADP) (Merck) was added to 50 µL of whole blood followed after 2 min by fixation with 1 mL of 1% paraformaldehyde (PFA) solution and incubation at 4°C overnight. The cells and platelets were spun down, washed in PBS, and finally resuspended in 1 mL of 2% Fetal Bovine Serum (FBS) solution (diluted in PBS). Then, 200 µL of the suspension was transferred to the flow cytometry tubes and the following antibodies were added: an anti-CD41 antibody conjugated with APC (2 µL/per tube, Invitrogen, clone HIP8, Thermofisher) was used to identify the entire platelet population, and an anti-CD62P antibody conjugated with BV421 (2 µl per tube, clone AK-4 304926, Biolegend) was used to identify the activated population. To detect TG2, a TG2 antibody conjugated with FITC (5 µL per tube, A028, Zedira) was used. Samples were measured on a BD FACS LSR II flow cytometer and analyzed with FlowJo software.

As shown in the figure below, the activation of platelets with 200 µM ADP was successful. As a non-activated control, gating was performed for the expected TG2 positive cells against CD41+. For platelets activated with ADP, the gating was performed against both CD41+ and CD62+. No TG2 was detected in CD41+ and CD62+ subpopulations.

**Addition to Figure 5. Turbidity curve obtained with plasma from a patient with FXIII inhibitor.** Absorbance values were normalized to the first time point and are displayed as mean and SEM.
