# Supplementary material for: The role of transglutaminase 2 in stabilizing fibrin clots and supporting hemostasis
Source: PLoS One. 2026 Aug 5;21(8):e0354080. doi: 10.1371/journal.pone.0354080 (PMC13440824; doi:10.1371/journal.pone.0354080)
Supplement: Supplementary file 2 [file pone.0354080.s003.docx]

**PONE-D-26-00575**

**Response to Reviewers**

The authors would like to thank all four reviewers for their positive feedback and very valuable and constructive criticism. We have taken your comments and suggestions into account and tried to improve our manuscript wherever possible. Please find below our replies (in blue).

**Reviewer #1**: The evaluated manuscript reports the results of an in vitro study addressing the potential role of tissue transglutaminase (TG2) in fibrin clot formation, stabilization and hemostasis. The formulated goals are timely, original and interesting. The study is based on adequate methodology. However, the major conclusion of the study that “TG2 promotes plasma clot formation, increases stability and prolongs lysis” is not fully supported by the reported data. Because of the identified limitations in the analysis of data, the interpretation of the results is not convincing. Thereby, the results are rather preliminary and larger sample sizes are needed to increase the statistical power of the analyses.

Major points

1. The relevance of the applied microfluidic bleeding model is not soundly supported by details of the geometry and rheometry of the applied device. A scheme of the microfluidic device with the dimensions of the channels and the flow rates is necessary to appreciate the relevance of the wall shear rates to the in vivo conditions. It is not clear what is the trigger of blood coagulation in the microfluidic device (the contact pathway is blocked with corn trypsin inhibitor, and no tissue factor is mentioned for this experimental setting). The presentation of the results from this model is also confusing; from the legend of Fig. 1D it is not clear what is the difference between the left and right half of the panel.

*Reply:* The group of our co-author Wilbur Lam (reference 26, Sakurai et al.) has characterized the bleeding model in great detail and provided detailed schemes on the geometry and wall shear stress. We have added additional information on the bleeding model to the text (chapter “Microfluidic bleeding model experiments” on pages 5-6) and refer again to the publication by Sakurai et al. [26]. The dimensions of the vascular channel are 150 μm in width and 50 μm in height, which are somewhat reduced and smoothened by the growth of the endothelial cell monolayer. These dimensions make the model comparable to human arterioles and venules. In this model, a flow rate of 2 μL/min corresponds to a shear rate of 500 s^-1^ which is comparable to a venous shear rate. Sakurai et al. [26] performed computational fluid dynamics simulations, which showed that, for the average wound size of 132.49 ± 40.19 μm, the initial shear stress at the middle of the wound site is approximately 3 dyne cm^−2^ (range: 2–5 dyne cm^−2^) and at the edge of wound approximately 7 dyne cm^−2^ (range: 4–8 dyne cm^−2^) when whole blood was perfused at 500 s^−1^.

As confirmed by Sakurai et al. [26], the trigger of blood coagulation is the mechanical injury causing disrupture of the endothelial cell monolayer and exposure of the subendothelial coating of collagen.

We have also amended the legend of Fig. 1 D (page 14): the panels on the left-hand side show the purple signal of the beta sheet domain TG2 antibody, the panels on the right-hand side show the purple signal of the core domain TG2 antibody.

2. Tables 1 and 2 report data originating from one or two ROTEM measurements – in case of two measurements only the mean is shown and not the two measured values which could provide some idea about the variance of the method. Clearly no statistical analysis could be performed on these data, and this questions the validity of the conclusions drawn from these measurements. Definitely larger sample size is necessary for the statistical evaluation of the ROTEM measurements. In Table 2, it is not clear why alpha-angle was assessed only for the autoimmune FXIII-deficient patient.

*Reply*: As ROTEM requires relatively large sample volumes (final volume 300 µL for each measurement), we were limited by the number of measurements we were able to perform. If we had sufficient funding available, we would increase the sample size, but this is unfortunately not possible. Nevertheless, despite of the small sample size and missing statistics, we believe the results do show a clear trend and argue for an effect of TG2 on clot formation.

We have now added the alpha angle to table 1 and where two measurements were performed we show not only mean values but all raw data.

3. For the evaluation of the turbidimetric curves, the authors use Dr Longstaff’s Shiny app. However, for this application a reliable zero point should be set for complete lysis. As shown in Fig. 4A, not all clots were completely lysed during the examined time interval. Thereby, these curves could not be correctly analysed with the cited app. A second major issue in this analysis is that the authors say “Clot lysis time was expressed as time from maximum absorbance to 50% clot lysis”. However, looking at the long plateau phase of the curves in Fig. 4A, any spike above the plateau line can be just a random variation of the measurement. So any random point from this plateau could be assigned as time of Amax and this makes the identification of the time for maximal absorbance uncertain. Definitely, measurements till complete lysis are necessary and objectively defined parameters of the lysis phase should be used. In view of these issues of the turbidimetric curve analysis in Fig. 4, the original traces for Fig. 5 should also be shown to support the validity of the analysis. Indeed, it is not clear why normalization of the original traces for the initial value was used in Fig. 4.

*Reply*: We have discussed the reviewer’s comments with Dr Colin Longstaff. He pointed out that it is very common in clot lysis assays to use the time to 50% lysis and it is not essential to use complete lysis as baseline. The reason 50% lysis is used when investigating clot lysis assays is that 100% lysis time and absorbance is often difficult to pin down precisely. It is common to see the baseline set to the readings at the beginning of the assay before clotting, which may be obvious and stable as the baseline. The app permits selection of such a baseline.

We agree with the reviewer that a long plateau around the maximum absorbance might make it difficult to establish a clear or correct time for maximum absorbance. We have therefore also analysed the time between 50% clotting and 50% lysis which is a usual way to express lysis time according to Dr Longstaff. We have added the additional graphs for figures 4 and 5 to the supporting information. These additional graphs show that TG2 also significantly prolongs time from 50% clotting to 50% lysis which is consistent with our results for the time from maximum absorbance to 50% lysis.

Normalization of the turbidity curves was used to show the curves starting at the same point which makes the curves easier to compare visually. It does not affect times to clotting or lysis.

We have now added to the methods paragraph some information about how we set the baseline for our measurements, why we used 50% lysis to analyse clot lysis, and about normalization (page 9), and we refer to the additional graphs in the results section (page 19).

4. In the experiments reported in Figs. 4 and 5 different plasmas were used – reference, commercial FXIII deficient, patient, but the levels of relevant factors that could affect the measurement (fibrinogen, coagulation factors and inhibitors) are not reported. Differences in plasma composition could influence the results. There is a controversy in the numeric values of maximal absorbance shown in panels A and C of Fig. 4.

*Reply*: The reference plasma contains coagulation factor levels including FXIII and fibrinogen in the normal range (confirmed in certificate of analysis; fibrinogen 2.83 g/L, FXIII 1 U/mL). According to the certificate of analysis of the commercial FXIII-depleted plasma, FXIII levels are <0.01 U/mL while other coagulation proteins and coagulation tests (PT, aPTT) are in the normal range. We have added this information to the text on page 9. In the patients’ samples, coagulation factor levels can of course vary. However, importantly, in our experiments we used the same material without and with addition of FXIII or TG2, so that we can compare the results within the same material and reliably assess the effect of the added transglutaminase.

Fig. 4, panels A and C: The panels show not the same, but normalized vs. relative absorbance values. In panel A, we wanted to directly compare the mean traces of the 4 different samples, therefore we normalized the curves, i.e. we set the absorbance at the starting point of all the traces to 1. In panel C, we show relative absorbance values in regard to the individual starting point of the measurement set as baseline for each individual curve. We have added this explanation to the figure legend (page 19).

5. In Table 3, it is strange that the relative prevalence of fibrinogen alpha chain is different from that of the beta and gamma chains as these are covalently attached. Maybe this is an indicator of the variance of the method? This discrepancy deserves some comments.

*Reply*: In Table 3 we compare the distribution of the detected crosslinked proteins between the plasma samples crosslinked with FXIII and the samples crosslinked with TG2. Since the transglutaminases crosslink the fibrin chains independently of each other, we cannot compare the distribution frequencies of the fibrin chains. The higher frequency of the fibrinogen alpha chain being crosslinked by FXIII is also consistent with the observation in purified fibrin clots that FXIII has a preference for crosslinking the fibrinogen alpha chain (Fig. 6C).

**Reviewer #2**: The current manuscript by Stoklosa et al. investigates the contribution of TG2 to fibrin cross-linking and clot stability. The authors also investigate the potential of TG2 to restore clot stability in the presence of FXIII autoantibodies. Although this approach could provide proof-of-concept for the therapeutic use of TG2 in FXIII deficiency, I have several major concerns which I feel should be addressed prior to publication.

• The number of replicates (n) for many assays is not clear, making it very challenging to assess the rigor and reproducibility of the findings. In Figures 1 and 2, the authors should indicate how many times the bleeding model and subsequent imaging were performed. Figure 2B-D indicates there are replicates, but it is not clear if a replicate consists of different passages of cells, different blood donors, or something else. Based on tables 1 and 2, it appears that the ROTEM experiment was only performed once. In figures 4 and 5, it is not clear if the listed n is referring to technical replicates, experiments performed on different days, or different batches of plasma. It is also not clear how many clots were analyzed for figure 6 and table 3.

*Reply*: We always used passage 5 endothelial cells for the microfluidic model (as we already stated in the text), but the cells were not always grown in the same flasks and at the same time. Blood donors were completely anonymous to us (as we stated in the text), but due to the large number of blood donations at the Blood Donation Center Bern we assume that they were different every time. For figure 1, we performed six individual experiments each with FXIII or TG2 peptide substrates, five individual experiments to detect endogenous TG2 protein, and five individual experiments to detect added recombinant TG2. ROTEM experiments were in part performed twice, but some were performed only once, as explained in the reply to comment 2 by reviewer 1.The turbidimetric assay was peformed always in duplicates (as stated in the methods part) and the number of experiments were individual experiments performed on different plates and/or days with the same materials and same badges of commercial plasmas. We analyzed two purified clots each crosslinked with either FXIII or TG2, and we analyzed seven plasma clots each made from normal plasma, FXIII-depleted plasma with FXIII and FXIII-depleted plasma with TG2.

Where this important imformation was missing or not clear, we have now added the number of replicates/experiments in the respective methods section or figure legend.

• For the imaging experiments in Figure 1A-B, the authors state in the methods that fibrin(ogen) is detected by incorporation of Alexa Fluor 488-labeled fibrinogen. However, the FXIII substrate (pepF11) is FITC-labeled. Because AF-488 and FITC have essentially the same ex/em spectrum, these fluorophores cannot be used simultaneously.

*Reply*: Thank you for pointing that out. We also used fibrinogen conjugated with Alexa Fluor™ 647, but forgot to mention that. We have now added this to the text on page 6.

• There are many instances where data presentation could be significantly improved. In figures 1 and 2, individual channels as well as merged images should be provided for all images, and scale bars should be included. It would be helpful to annotate the images so that the reader can identify the wound and where the "bleeding" is occuring. In figure 2D, the authors report that there is no difference in fibrinogen staining between the three groups, but the quantitation does not reflect what is shown in the representative images. The area of intense green fluorescence in the whole blood + ZED1301 group appears to be much smaller than what is shown in the other two groups, so the authors should provide clarification on how positive pixels were identified. Finally, in figures 4 and 5, it is not clear what the dotted lines connecting the points indicate.

*Reply*: We thank the reviewer for this comment, however, we believe it would exceed the possible and also meaningful amount of figures if we always showed all individual channels, so we have carefully chosen representative figures. Scale bars are included but we agree that they are not well visible in some instances. We will ask for support from the graphics experts when the layout of the manuscript is produced. We are not quite sure what the reviewer means by “annotating the images”, as the wound and bleeding area is clearly visible on the images in our opinion. The green fluorescence signal for fibrinogen/fibrin does not differ between groups and as shown in figure 2D was highest in the whole blood only group. The important point in figure 2 is the purple signal for fibrin crosslinks which is clearly lower in the WB and ZED1301 FXIII inhibitor group but higher in whole blood (effect of FXIII) and the TG2 group even in presence of FXIII inhibitor (effect of TG2). The dotted lines in figures 4 and 5 indicate direct comparisons between paired samples, we have added this information to the figure legends.

• The physiologic relevance of the system used for the cross-linking mass spec experiments is not clear. Normal human plasma fibrinogen concentration is typically 2-4 mg/mL. This design makes the fibrinogen concentration abnormally high (10 mg/ml), but the FXIII concentration close to normal levels (70 nM).

*Reply*: In the purified system, when using only purified fibrinogen and FXIII or TG2 with thrombin and Ca^2+^ to induce fibrin clot formation, we need a higher concentration of fibrinogen as the only matrix protein to get a clot that is stable enough for washing. We agree that this concentration does not reflect physiological levels. However, a purified system has always limited physiological relevance. Therefore, we always try and combine experiments in purifed systems with experiments in plasma (or even whole blood in case of the microfluidic model) which are closer to physiological conditions (but still an artificial in vitro system, of course). These limitations always need to be taken into account when interpreting the results.

• Relevant experimental controls should not be omitted from the results. The authors should show the data showing that the TG2 inhibitor (Z006) inhibits the incorporation of the TAMRA-T26 substrate (currently not shown). There are other cases were relevant controls should be included. For instance, the authors report that recombinant TG2 can be detected with the TG2 antibodies, but activity is not shown. This would provide important validation of the findings. The authors should also demonstrate that the DD-XLink-mAb staining in the whole blood + ZED1301 + TG2 group is TG2-dependent using the TG2 inhibitor.

*Reply*: As suggested, we have added the control experiments with the control peptide with abolished crosslinking site (FITC-F11QN) and the inhibitor Z006 to the supporting information. We have chosen a combination of functional detection using substrate peptides and antigen detection using antibodies. Due to limited availability of materials we had to compromise and are unfortunately not able to repeat all the experiments always with all settings and conditions. We are still convinced that our results do sufficiently show both effects of TG2 activity and antigen or FXIII activity and antigen.

• The fibrinogen concentrations for the reference plasma and FXIII-deficient plasma used in the turbidity experiments should be reported because differences in fibrinogen concentration drastically alter turbidity measurements.

*Reply*: The reference plasma contains coagulation factor levels including FXIII and fibrinogen in the normal range (confirmed in certificate of analysis; fibrinogen 2.83 g/L, FXIII 1 U/mL). According to the certificate of analysis of the commercial FXIII-depleted plasma, FXIII levels are <0.01 U/mL while other coagulation proteins and coagulation tests (PT, aPTT) are in the normal range. We have added this information to the text on page 9. However, importantly, in our experiments we used the same material without and with addition of FXIII or TG2, so that we can compare the results within the same material and reliably assess the effect of the added transglutaminase.

**Reviewer #3**: This manuscript investigates whether transglutaminase 2 (TG2) can contribute to fibrin crosslinking and clot stabilization, particularly in the absence or inhibition of FXIII. The study addresses an important and clinically relevant question, especially in the context of autoimmune acquired FXIII deficiency, where therapeutic options are limited. The combination of functional assays, a physiologically relevant microfluidic bleeding model, and proteomic analyses represents a clear strength of the work. The microfluidic endothelialized bleeding model is particularly compelling. The visualization of TG2 activity at the injury site and the demonstration that exogenous TG2 restores fibrin crosslinking in the presence of an FXIII inhibitor provide strong proof-of-principle evidence. The consistency between imaging data and functional assays (ROTEM and turbidity) supports the central conclusion that TG2 can enhance clot stability and delay fibrinolysis. However, several aspects require clarification or strengthening:

First, statistical transparency should be improved. The manuscript does not consistently state the number of independent biological replicates for each experiment. In some cases (e.g., ROTEM Table 1), mean values from two experiments are shown, which limits statistical robustness. Please clearly indicate n numbers for all datasets, describe whether data distribution was assessed before applying parametric tests, and clarify how multiple comparisons were handled. This will increase confidence in the reported significance.

*Reply*: For the microfluidic model experiments shown in figure 1, we performed six individual experiments each with FXIII or TG2 peptide substrates, five individual experiments to detect endogenous TG2 protein, and five individual experiments to detect added recombinant TG2.

ROTEM experiments were in part performed twice, but some were performed only once. As ROTEM requires relatively large sample volumes (final volume 300 µL for each measurement), we were limited by the number of measurements we were able to perform. If we had sufficient funding available, we would increase the sample size, but this is unfortunately not possible. Nevertheless, despite of the small sample size and missing statistics, we believe the results do show a clear trend and argue for an effect of TG2 on clot formation.

The turbidimetric assay was peformed always in duplicates (as stated in the methods part) and the number of experiments were individual experiments performed on different plates and/or days with the same materials and same badges of commercial plasmas. We analyzed two purified clots each crosslinked with either FXIII or TG2, and we analyzed seven plasma clots each made from normal plasma, FXIII-depleted plasma with FXIII and FXIII-depleted plasma with TG2.

Where this important imformation was missing or not clear, we have now added the number of replicates/experiments in the respective methods section or figure legend.

Second, the physiological relevance of endogenous TG2 at the injury site remains somewhat uncertain. The manuscript acknowledges variability in detection of endogenous TG2 in the microfluidic model. It would strengthen the paper to quantify how often TG2 was detectable across independent donor experiments and to provide a more structured discussion of possible sources (endothelium, erythrocytes, hemolysis, etc.). At present, the data strongly support the activity of exogenously added TG2, but the role of endogenous TG2 in normal hemostasis is less definitively demonstrated.

*Reply*: We have added the number of independent donor experiments (in total n=5) to the methods section on page 7. Over all the experiments (n=5), TG2 was detectable five times (three times with the beta sheet domain antibody and twice with the core domain antibody), and it was not detectable three times (once with the beta sheet antibody and twice with the core domain antibody). We have added this information to the results section on page 13. We have not extended the discussion, as we feel that at this moment we could only provide further speculations which would not be well supported. Further experiments into possible endogenous sources of TG2 are planned for a follow-up project.

Third, the autoimmune FXIII deficiency experiments are interesting but require more careful interpretation. The observation that recombinant FXIII-A2 improved some parameters even in the presence of autoantibodies raises questions about inhibitor strength and neutralization efficiency. Please clarify whether inhibitor titers were sufficient to fully block added FXIII-A2 under the experimental conditions. Additionally, the possibility of partial inhibition or differential sensitivity in ROTEM versus turbidity assays should be discussed. Since the therapeutic concept is central to the manuscript, these mechanistic ambiguities should be addressed more directly.

*Reply*: As already described in the text (pages 8 and 9), no FXIII activity and no FXIII antigen or <2% of FXIII antigen were detectable in the patient’s samples used in our experiments, and the amount of inhibitor was determined as 8, 10 and 20 Bethesda Units. One Bethesda unit is defined as the amount of inhibitor that neutralizes 50% of the coagulation factor activity in 1 mL of normal plasma after 2 hours of incubation at 37°C, so the inhibitor titers measured in the samples are expected to fully neutralize any FXIII. We fully agree that the therapeutic concept needs to be confirmed in further and more detailed experiments. We have highlighted the pilot character of our study in the discussion section on page 26.

Fourth, the proteomic and crosslinking data are valuable and add depth to the manuscript. The distinct crosslinking patterns and differential incorporation of plasma proteins into TG2- versus FXIII-generated clots are intriguing findings. However, more methodological detail would be helpful: number of biological replicates analyzed, FDR thresholds, and whether raw MS data will be deposited in a public proteomics repository. These details are important for reproducibility.

*Reply*: We have added the number of replicates (two clots each made from purified proteins and seven clots each made from plasma) to the text on page 10. The false discovery rate thresholds were set at 1%, this information was already given in the detailed methods description in the supporting information. In a follow-up project we will analyze the crosslinking sites in more detail and will then also deposit the raw MS data in a public repository.

Finally, the therapeutic framing should be slightly moderated. The manuscript convincingly demonstrates that TG2 can stabilize fibrin clots in vitro and in ex vivo systems. However, systemic administration of TG2 would raise significant safety considerations, including off-target crosslinking and potential prothrombotic effects. It would strengthen the discussion to emphasize that the current findings represent proof-of-concept and that substantial in vivo validation would be required before clinical translation.

*Reply*: We fully agree that the scope of this manuscript was merely proof-of-concept that TG2 can actually stabilize fibrin clots. The motivation for our study was in the context of (autoimmune) FXIII deficiency. But of course, further experimental in vitro and in vivo studies are essential before studies for any clinical translation could be envisaged. As suggested, we have emphasized this even more in the discussion section on pages 26-27.

In summary, this is a well-designed and innovative study with strong experimental breadth. With improved statistical clarity, deeper discussion of inhibitor experiments, expanded methodological detail for proteomics, and slightly more cautious therapeutic interpretation, the manuscript would make a meaningful contribution to the field of coagulation and fibrin biology.

**Reviewer #4**: This article is very well written, with a clear experimental design, and focuses on a topic that is increasingly at the forefront of coagulation research. While not fully understood, research into better understanding the differences between FXIII and TG2 is helping to elucidate future treatments for patients with severe bleeding disorders.

I believe the article should be published as is, but I wonder whether additional clot structure images would help better understand the differences found between FXIII and TG2 during turbidity and lysis experiments. The authors even suggest these experiments in the discussion, stating, " ... TG2 plasma clots showed lower turbidity which may be a result of different clot architecture or protein composition." Fluorescent images of fully formed clots would be straightforward and would add additional insight into the differences observed between viscoelastic and turbidimetric experiments.

*Reply:* Thank you for your extremely positive feedback. We agree with the reviewer that clot structure images would be of great interest. Unfortunately, we have currently no funding to conduct the experiments. We have applied for funding for a follow-up project and extensive clot structure analysis (by scanning electron microscopy and confocal microscopy) will be part of this follow-up project if funded.
