# Supplementary material for: The role of transglutaminase 2 in stabilizing fibrin clots and supporting hemostasis
Source: PLoS One. 2026 Aug 5;21(8):e0354080. doi: 10.1371/journal.pone.0354080 (PMC13440824; doi:10.1371/journal.pone.0354080)
Supplement: Supplementary file 3 [file pone.0354080.s004.docx]

**PONE-D-26-00575R1**

**Response to Reviewers**

Please find below our changes made during this second revision and replies to the reviewers’ comments.

**Reviewer #1:** In the revised manuscript, the authors have addressed some of the critiques raised, but several critical issues remain inadequately addressed.

Major points

1. Responding to the original comment “It is not clear what is the trigger of blood coagulation in the microfluidic device (the contact pathway is blocked with corn trypsin inhibitor, and no tissue factor is mentioned for this experimental setting)”, the authors state that “the trigger of blood coagulation is the mechanical injury causing disruption of the endothelial cell monolayer and exposure of the subendothelial coating of collagen”. It remains unclear how mechanical injury and collagen exposure can trigger clotting when the contact pathway is blocked, and no tissue factor is present in the experimental setup used in this study.

*Reply:* As explained in detail in the first revision, the bleeding model used here was developed and thoroughly characterised and described by the group of Prof. Wilbur Lam, Atlanta, USA (Sakurai et al. [26]). In their publication, they also demonstrated the process of blood coagulation activation: “As expected, upon mechanical introduction of the wound, blood not only continues to perfuse the vascular channel (Fig. 1c, horizontal flow) but also flows into the newly formed wound channel, analogous to “bleeding” (Fig. 1c, vertical flow). As blood continues to flow through the vascular and wound channels, platelets adhere throughout the injury site, platelet aggregation leads to the formation of a hemostatic plug at the wound site, fibrin polymerization occurs, and finally, cessation of blood flow is achieved (Fig. 1e, Supplementary Movie 1).” [26] Therefore, it is not the scope of this manuscript to repeat what has been shown by Sakurai et al. [26] and we trust that the same process also occurs in our experiments: ruptured endothelial cells release von Willebrand factor and subendothelial collagen is exposed, platelets adhere to both von Willebrand factor bound to collagen and to collagen directly, get activated, secrete intracellular procoagulant proteins, expose receptors such as integrins and negatively charged phospholipids on the platelet surface (“primary haemostasis”), which all leads to activation of the blood coagulation cascade with binding and activation of coagulation factors by complex assembly on the platelet surface and eventually fibrin formation and stabilisation (“secondary haemostasis”).

When we tried to run the model with platelet poor plasma instead of whole blood, it did not clot, so platelets were necessary, which is some additional experimental evidence that coagulation is activated via primary haemostasis.

We have added some additional explanation to the text:

Page 6: As described by Sakurai et al. [26], the trigger of blood coagulation is the mechanical injury causing disrupture of the endothelial cell monolayer - leading to release of von Willebrand factor - and exposure of the subendothelial coating of collagen - leading to platelet adhesion and activation and formation of a platelet plug (primary hemostasis) which also activates the coagulation cascade leading to fibrin formation (secondary hemostasis).

2. In response to the original critique regarding the sample size of the ROTEM measurements, the authors state that “despite the small sample size and missing statistics, we believe the results do show a clear trend and argue for an effect of TG2 on clot formation”. However, the lack of funds does not justify including these preliminary results, as their interpretation is not adequately supported and could be misleading. For example, referring to Fig. 3 and the numeric parameters reported in Table 1, the authors claim a dose-dependent effect of TG2 on MCF. However, if measurements from a larger sample show that one of the two values currently reported for 40 µg/mL TG2 (12 and 18) is an outlier, then the MCF at all 3 doses would be the same, and the current conclusion would be invalid. Therefore, the small-sample ROTEM data should be excluded from this report if sufficiently large samples cannot be measured.

*Reply:* Although the other reviewers did not criticise the ROTEM experiments, we have now removed them from the manuscript as demanded by reviewer 1.

3. Regarding the analysis of the turbidimetric curves, the authors state that “it is not essential to use complete lysis as baseline”. I agree with this view if the turbidity at the start and end of the process is the same. This generally holds for clot lysis of pure fibrin, but not always for more complex plasma clots. This issue is clearly illustrated by the curves in the supplementary Figure 5. How can the authors interpret values below 1 at the end of lysis if they use the difference between the start-point absorbance and the maximal absorbance to estimate 50% lysis? I also agree that the time from 50% clotting to 50% lysis is a reliable estimate of lytic efficiency. If the authors acknowledge this, they should replace all figures showing the time from Amax (which is rather arbitrary) to 50% lysis with those added to the revised supplement.

*Reply:* We have now moved the figures from the supplement to the figures in the main manuscript and replaced lysis time expressed from maximum absorbance to 50% lysis by lysis time expressed from 50% clotting to 50% lysis.

**Reviewer #2:** I find the revised manuscript to be significantly improved, and I thank the authors for taking the time to address my comments. I have only minor comments regarding data presentation which I believe should be considered prior to publication.

*Reply:* Thank you very much for your positive feedback.

• For clarity, it would be helpful if details about the number of replicates were added to the figure legend in addition to the methods. For example, the legend for figure 1 could include “Experiments were performed with blood from 6 unique donors (n=6),” and the legend for the turbidity experiments could include “Experiments were performed in 5 unique replicates with the same batches of plasma (n=5). ”

*Reply:* As suggested, we have included the information on number of experiments in the legend for figure 1 and the legends for the figures showing the turbidity experiments.

• Unless they are just extremely difficult to see, several microscopy images still appear to be lacking scale bars (Fig. 1A, 1B, bottom two panels of 1D). In other images (e.g., Fig 1D top set of images), a scale bar is included, but it does not appear to be indicated anywhere what distance the scale bar corresponds to.

*Reply:* As suggested, we have added the missing scale bars.

• In Figure 4 B-D, the phrase “dotted lines indicate direct comparisons between paired samples,” is somewhat misleading. Because these are two different sources of plasma, they cannot truly be “paired.” I fully agree, however, that this is an appropriate comparison for the other three groups (FXIII-deficient plasma with TG2 or FXIII). I believe it would be most appropriate to remove the dotted line between the black symbols and pink symbols. If the authors wish to include the reference plasma from the same experimental replicate for comparison, they could consider using different shapes for the symbols or something similar.

*Reply:* As suggested, we have removed the dotted lines.

**Reviewer #3:** I thank the authors for their thorough responses to the previous review comments and for the substantial revisions made to the manuscript. The revised version provides a significantly strengthened and more balanced assessment of the potential role of transglutaminase 2 (TG2) in fibrin clot stabilization. The combination of a microfluidic endothelialized bleeding model, viscoelastic analyses, clot formation and lysis assays, and proteomic characterization provides a coherent body of evidence supporting the conclusion that TG2 is capable of contributing to fibrin crosslinking and clot stabilization. Importantly, the authors have appropriately acknowledged the limitations of the study, particularly regarding the physiological relevance of endogenous TG2 and the preliminary nature of the therapeutic implications. The conclusions are now generally well aligned with the presented data. The proteomic analyses revealing distinct crosslinking patterns and differential protein incorporation between TG2-mediated and FXIII-mediated clots add further value to the manuscript and provide a useful basis for future mechanistic studies. Only minor typographical and language issues remain and can be addressed during final editing. Overall, I believe the manuscript is technically sound, the conclusions are supported by the data presented, and the work will be of interest to researchers studying coagulation, fibrin clot biology, and transglutaminase function. I support publication in PLOS ONE.

*Reply:* Thank you very much for your positive feedback. We have thoroughly checked the manuscript again and corrected some typographical errors.

**Reviewer #4:** The authors have addressed all comments and suggestions. I believe that the current state of the manuscript is sound with added information from the other reviewers and am happy for it to published in it's current state.

*Reply:* Thank you again for your positive feedback.
