# Supplementary material for: The role of transglutaminase 2 in stabilizing fibrin clots and supporting hemostasis
Source: PLoS One. 2026 Aug 5;21(8):e0354080. doi: 10.1371/journal.pone.0354080 (PMC13440824; doi:10.1371/journal.pone.0354080)
Supplement: Peer review history

# Peer review: Decision Letter 0

**DOI:** 10.1371/journal.pone.0354080.r001

**Authors:** Osman El-Maarri

11 Mar 2026

PONE-D-26-00575

The role of transglutaminase 2 in stabilizing fibrin clots and supporting hemostasis

PLOS One

Dear Dr. Schroeder,

Thank you for submitting your manuscript to PLOS ONE. After careful consideration, we feel that it has merit but does not fully meet PLOS ONE’s publication criteria as it currently stands. Therefore, we invite you to submit a revised version of the manuscript that addresses the points raised during the review process.

Please submit your revised manuscript by Apr 25 2026 11:59PM. If you will need more time than this to complete your revisions, please reply to this message or contact the journal office at plosone@plos.org. When you're ready to submit your revision, log on to https://www.editorialmanager.com/pone/ and select the 'Submissions Needing Revision' folder to locate your manuscript file.

Please include the following items when submitting your revised manuscript:

- A letter that responds to each point raised by the academic editor and reviewer(s). You should upload this letter as a separate file labeled 'Response to Reviewers'.
- A marked-up copy of your manuscript that highlights changes made to the original version. You should upload this as a separate file labeled 'Revised Manuscript with Track Changes'.
- An unmarked version of your revised paper without tracked changes. You should upload this as a separate file labeled 'Manuscript'.

If you would like to make changes to your financial disclosure, please include your updated statement in your cover letter. Guidelines for resubmitting your figure files are available below the reviewer comments at the end of this letter.

If applicable, we recommend that you deposit your laboratory protocols in protocols.io to enhance the reproducibility of your results. Protocols.io assigns your protocol its own identifier (DOI) so that it can be cited independently in the future. For instructions see: https://journals.plos.org/plosone/s/submission-guidelines#loc-laboratory-protocols. Additionally, PLOS ONE offers an option for publishing peer-reviewed Lab Protocol articles, which describe protocols hosted on protocols.io. Read more information on sharing protocols at https://plos.org/protocols?utm_medium=editorial-email&utm_source=authorletters&utm_campaign=protocols.

We look forward to receiving your revised manuscript.

Kind regards,

Osman El-Maarri, Ph.D

Academic Editor

PLOS One

Journal requirements:

When submitting your revision, we need you to address these additional requirements.

1. Please ensure that your manuscript meets PLOS ONE's style requirements, including those for file naming. The PLOS ONE style templates can be found at

https://journals.plos.org/plosone/s/file?id=wjVg/PLOSOne_formatting_sample_main_body.pdf and

https://journals.plos.org/plosone/s/file?id=ba62/PLOSOne_formatting_sample_title_authors_affiliations.pdf

2. When completing the data availability statement of the submission form, you indicated that you will make your data available on acceptance. We strongly recommend all authors decide on a data sharing plan before acceptance, as the process can be lengthy and hold up publication timelines. Please note that, though access restrictions are acceptable now, your entire data will need to be made freely accessible if your manuscript is accepted for publication. This policy applies to all data except where public deposition would breach compliance with the protocol approved by your research ethics board. If you are unable to adhere to our open data policy, please kindly revise your statement to explain your reasoning and we will seek the editor's input on an exemption. Please be assured that, once you have provided your new statement, the assessment of your exemption will not hold up the peer review process.

If the reviewer comments include a recommendation to cite specific previously published works, please review and evaluate these publications to determine whether they are relevant and should be cited. There is no requirement to cite these works unless the editor has indicated otherwise.

[Note: HTML markup is below. Please do not edit.]

Reviewers' comments:

Reviewer's Responses to Questions

**Comments to the Author**

1. Is the manuscript technically sound, and do the data support the conclusions?

The manuscript must describe a technically sound piece of scientific research with data that supports the conclusions. Experiments must have been conducted rigorously, with appropriate controls, replication, and sample sizes. The conclusions must be drawn appropriately based on the data presented.

Reviewer #1: No

Reviewer #2: No

Reviewer #3: Yes

Reviewer #4: Yes

**********

2. Has the statistical analysis been performed appropriately and rigorously?

Reviewer #1: No

Reviewer #2: No

Reviewer #3: Yes

Reviewer #4: Yes

**********

3. Have the authors made all data underlying the findings in their manuscript fully available?

The PLOS Data policy requires authors to make all data underlying the findings described in their manuscript fully available without restriction, with rare exception (please refer to the Data Availability Statement in the manuscript PDF file). The data should be provided as part of the manuscript or its supporting information, or deposited to a public repository. For example, in addition to summary statistics, the data points behind means, medians and variance measures should be available. If there are restrictions on publicly sharing data—e.g. participant privacy or use of data from a third party—those must be specified.

Reviewer #1: No

Reviewer #2: No

Reviewer #3: Yes

Reviewer #4: Yes

**********

4. Is the manuscript presented in an intelligible fashion and written in standard English?

PLOS ONE does not copyedit accepted manuscripts, so the language in submitted articles must be clear, correct, and unambiguous. Any typographical or grammatical errors should be corrected at revision, so please note any specific errors here.

Reviewer #1: Yes

Reviewer #2: Yes

Reviewer #3: Yes

Reviewer #4: Yes

**********

5. Review Comments to the Author

Please use the space provided to explain your answers to the questions above. You may also include additional comments for the author, including concerns about dual publication, research ethics, or publication ethics. (Please upload your review as an attachment if it exceeds 20,000 characters)

Reviewer #1: The evaluated manuscript reports the results of an in vitro study addressing the potential role of tissue transglutaminase (TG2) in fibrin clot formation, stabilization and hemostasis. The formulated goals are timely, original and interesting. The study is based on adequate methodology. However, the major conclusion of the study that “TG2 promotes plasma clot formation, increases stability and prolongs lysis” is not fully supported by the reported data. Because of the identified limitations in the analysis of data, the interpretation of the results is not convincing. Thereby, the results are rather preliminary and larger sample sizes are needed to increase the statistical power of the analyses.

Major points

1. The relevance of the applied microfluidic bleeding model is not soundly supported by details of the geometry and rheometry of the applied device. A scheme of the microfluidic device with the dimensions of the channels and the flow rates is necessary to appreciate the relevance of the wall shear rates to the in vivo conditions. It is not clear what is the trigger of blood coagulation in the microfluidic device (the contact pathway is blocked with corn trypsin inhibitor, and no tissue factor is mentioned for this experimental setting). The presentation of the results from this model is also confusing; from the legend of Fig. 1D it is not clear what is the difference between the left and right half of the panel.

2. Tables 1 and 2 report data originating from one or two ROTEM measurements – in case of two measurements only the mean is shown and not the two measured values which could provide some idea about the variance of the method. Clearly no statistical analysis could be performed on these data, and this questions the validity of the conclusions drawn from these measurements. Definitely larger sample size is necessary for the statistical evaluation of the ROTEM measurements. In Table 2, it is not clear why alpha-angle was assessed only for the autoimmune FXIII-deficient patient.

3. For the evaluation of the turbidimetric curves, the authors use Dr Longstaff’s Shiny app. However, for this application a reliable zero point should be set for complete lysis. As shown in Fig. 4A, not all clots were completely lysed during the examined time interval. Thereby, these curves could not be correctly analysed with the cited app. A second major issue in this analysis is that the authors say “Clot lysis time was expressed as time from maximum absorbance to 50% clot lysis”. However, looking at the long plateau phase of the curves in Fig. 4A, any spike above the plateau line can be just a random variation of the measurement. So any random point from this plateau could be assigned as time of Amax and this makes the identification of the time for maximal absorbance uncertain. Definitely, measurements till complete lysis are necessary and objectively defined parameters of the lysis phase should be used. In view of these issues of the turbidimetric curve analysis in Fig. 4, the original traces for Fig. 5 should also be shown to support the validity of the analysis. Indeed, it is not clear why normalization of the original traces for the initial value was used in Fig. 4.

4. In the experiments reported in Figs. 4 and 5 different plasmas were used – reference, commercial FXIII deficient, patient, but the levels of relevant factors that could affect the measurement (fibrinogen, coagulation factors and inhibitors) are not reported. Differences in plasma composition could influence the results. There is a controversy in the numeric values of maximal absorbance shown in panels A and C of Fig. 4.

5. In Table 3, it is strange that the relative prevalence of fibrinogen alpha chain is different from that of the beta and gamma chains as these are covalently attached. Maybe this is an indicator of the variance of the method? This discrepancy deserves some comments.

Reviewer #2: The current manuscript by Stoklosa et al. investigates the contribution of TG2 to fibrin cross-linking and clot stability. The authors also investigate the potential of TG2 to restore clot stability in the presence of FXIII autoantibodies. Although this approach could provide proof-of-concept for the therapeutic use of TG2 in FXIII deficiency, I have several major concerns which I feel should be addressed prior to publication.

• The number of replicates (n) for many assays is not clear, making it very challenging to assess the rigor and reproducibility of the findings. In Figures 1 and 2, the authors should indicate how many times the bleeding model and subsequent imaging were performed. Figure 2B-D indicates there are replicates, but it is not clear if a replicate consists of different passages of cells, different blood donors, or something else. Based on tables 1 and 2, it appears that the ROTEM experiment was only performed once. In figures 4 and 5, it is not clear if the listed n is referring to technical replicates, experiments performed on different days, or different batches of plasma. It is also not clear how many clots were analyzed for figure 6 and table 3.

• For the imaging experiments in Figure 1A-B, the authors state in the methods that fibrin(ogen) is detected by incorporation of Alexa Fluor 488-labeled fibrinogen. However, the FXIII substrate (pepF11) is FITC-labeled. Because AF-488 and FITC have essentially the same ex/em spectrum, these fluorophores cannot be used simultaneously.

• There are many instances where data presentation could be significantly improved. In figures 1 and 2, individual channels as well as merged images should be provided for all images, and scale bars should be included. It would be helpful to annotate the images so that the reader can identify the wound and where the "bleeding" is occuring. In figure 2D, the authors report that there is no difference in fibrinogen staining between the three groups, but the quantitation does not reflect what is shown in the representative images. The area of intense green fluorescence in the whole blood + ZED1301 group appears to be much smaller than what is shown in the other two groups, so the authors should provide clarification on how positive pixels were identified. Finally, in figures 4 and 5, it is not clear what the dotted lines connecting the points indicate.

• The physiologic relevance of the system used for the cross-linking mass spec experiments is not clear. Normal human plasma fibrinogen concentration is typically 2-4 mg/mL. This design makes the fibrinogen concentration abnormally high (10 mg/ml), but the FXIII concentration close to normal levels (70 nM).

• Relevant experimental controls should not be omitted from the results. The authors should show the data showing that the TG2 inhibitor (Z006) inhibits the incorporation of the TAMRA-T26 substrate (currently not shown). There are other cases were relevant controls should be included. For instance, the authors report that recombinant TG2 can be detected with the TG2 antibodies, but activity is not shown. This would provide important validation of the findings. The authors should also demonstrate that the DD-XLink-mAb staining in the whole blood + ZED1301 + TG2 group is TG2-dependent using the TG2 inhibitor.

• The fibrinogen concentrations for the reference plasma and FXIII-deficient plasma used in the turbidity experiments should be reported because differences in fibrinogen concentration drastically alter turbidity measurements.

Reviewer #3: This manuscript investigates whether transglutaminase 2 (TG2) can contribute to fibrin crosslinking and clot stabilization, particularly in the absence or inhibition of FXIII. The study addresses an important and clinically relevant question, especially in the context of autoimmune acquired FXIII deficiency, where therapeutic options are limited. The combination of functional assays, a physiologically relevant microfluidic bleeding model, and proteomic analyses represents a clear strength of the work. The microfluidic endothelialized bleeding model is particularly compelling. The visualization of TG2 activity at the injury site and the demonstration that exogenous TG2 restores fibrin crosslinking in the presence of an FXIII inhibitor provide strong proof-of-principle evidence. The consistency between imaging data and functional assays (ROTEM and turbidity) supports the central conclusion that TG2 can enhance clot stability and delay fibrinolysis. However, several aspects require clarification or strengthening:

First, statistical transparency should be improved. The manuscript does not consistently state the number of independent biological replicates for each experiment. In some cases (e.g., ROTEM Table 1), mean values from two experiments are shown, which limits statistical robustness. Please clearly indicate n numbers for all datasets, describe whether data distribution was assessed before applying parametric tests, and clarify how multiple comparisons were handled. This will increase confidence in the reported significance.

Second, the physiological relevance of endogenous TG2 at the injury site remains somewhat uncertain. The manuscript acknowledges variability in detection of endogenous TG2 in the microfluidic model. It would strengthen the paper to quantify how often TG2 was detectable across independent donor experiments and to provide a more structured discussion of possible sources (endothelium, erythrocytes, hemolysis, etc.). At present, the data strongly support the activity of exogenously added TG2, but the role of endogenous TG2 in normal hemostasis is less definitively demonstrated.

Third, the autoimmune FXIII deficiency experiments are interesting but require more careful interpretation. The observation that recombinant FXIII-A2 improved some parameters even in the presence of autoantibodies raises questions about inhibitor strength and neutralization efficiency. Please clarify whether inhibitor titers were sufficient to fully block added FXIII-A2 under the experimental conditions. Additionally, the possibility of partial inhibition or differential sensitivity in ROTEM versus turbidity assays should be discussed. Since the therapeutic concept is central to the manuscript, these mechanistic ambiguities should be addressed more directly.

Fourth, the proteomic and crosslinking data are valuable and add depth to the manuscript. The distinct crosslinking patterns and differential incorporation of plasma proteins into TG2- versus FXIII-generated clots are intriguing findings. However, more methodological detail would be helpful: number of biological replicates analyzed, FDR thresholds, and whether raw MS data will be deposited in a public proteomics repository. These details are important for reproducibility.

Finally, the therapeutic framing should be slightly moderated. The manuscript convincingly demonstrates that TG2 can stabilize fibrin clots in vitro and in ex vivo systems. However, systemic administration of TG2 would raise significant safety considerations, including off-target crosslinking and potential prothrombotic effects. It would strengthen the discussion to emphasize that the current findings represent proof-of-concept and that substantial in vivo validation would be required before clinical translation.

In summary, this is a well-designed and innovative study with strong experimental breadth. With improved statistical clarity, deeper discussion of inhibitor experiments, expanded methodological detail for proteomics, and slightly more cautious therapeutic interpretation, the manuscript would make a meaningful contribution to the field of coagulation and fibrin biology.

Reviewer #4: This article is very well written, with a clear experimental design, and focuses on a topic that is increasingly at the forefront of coagulation research. While not fully understood, research into better understanding the differences between FXIII and TG2 is helping to elucidate future treatments for patients with severe bleeding disorders.

I believe the article should be published as is, but I wonder whether additional clot structure images would help better understand the differences found between FXIII and TG2 during turbidity and lysis experiments. The authors even suggest these experiments in the discussion, stating, " ... TG2 plasma clots showed lower turbidity which may be a result of different clot architecture or protein composition." Fluorescent images of fully formed clots would be straightforward and would add additional insight into the differences observed between viscoelastic and turbidimetric experiments.

**********

6. PLOS authors have the option to publish the peer review history of their article (what does this mean?). If published, this will include your full peer review and any attached files.

If you choose “no”, your identity will remain anonymous but your review may still be made public.

**Do you want your identity to be public for this peer review?**  For information about this choice, including consent withdrawal, please see our Privacy Policy.

Reviewer #1: No

Reviewer #2: No

Reviewer #3: **Yes:**  Arijit Biswas

Reviewer #4: No

**********

[NOTE: If reviewer comments were submitted as an attachment file, they will be attached to this email and accessible via the submission site. Please log into your account, locate the manuscript record, and check for the action link "View Attachments". If this link does not appear, there are no attachment files.]

To ensure your figures meet our technical requirements, please review our figure guidelines: https://journals.plos.org/plosone/s/figures

You may also use PLOS’s free figure tool, NAAS, to help you prepare publication quality figures: https://journals.plos.org/plosone/s/figures#loc-tools-for-figure-preparation.

NAAS will assess whether your figures meet our technical requirements by comparing each figure against our figure specifications.

# Author comment: Author response to Decision Letter 1

**DOI:** 10.1371/journal.pone.0354080.r002

13 May 2026

I have responded to all comments in the document "Response to Reviewers".

Attachment Submitted filename: Response to Reviewers.docx

# Peer review: Decision Letter 1

**DOI:** 10.1371/journal.pone.0354080.r003

**Authors:** Osman El-Maarri

3 Jun 2026

PONE-D-26-00575R1

The role of transglutaminase 2 in stabilizing fibrin clots and supporting hemostasis

PLOS One

Dear Dr. Schroeder,

Thank you for submitting your manuscript to PLOS ONE. After careful consideration, we feel that it has merit but does not fully meet PLOS ONE’s publication criteria as it currently stands. Therefore, we invite you to submit a revised version of the manuscript that addresses the points raised during the review process.

=============================

**Therefore before we issue formal acceptance we ask you to make final minor editing.**

=============================

Please submit your revised manuscript by Jul 18 2026 11:59PM. If you will need more time than this to complete your revisions, please reply to this message or contact the journal office at plosone@plos.org. When you're ready to submit your revision, log on to https://www.editorialmanager.com/pone/ and select the 'Submissions Needing Revision' folder to locate your manuscript file.

Please include the following items when submitting your revised manuscript:

- A letter that responds to each point raised by the academic editor and reviewer(s). You should upload this letter as a separate file labeled 'Response to Reviewers'.
- A marked-up copy of your manuscript that highlights changes made to the original version. You should upload this as a separate file labeled 'Revised Manuscript with Track Changes'.
- An unmarked version of your revised paper without tracked changes. You should upload this as a separate file labeled 'Manuscript'.

If you would like to make changes to your financial disclosure, please include your updated statement in your cover letter. Guidelines for resubmitting your figure files are available below the reviewer comments at the end of this letter.

If applicable, we recommend that you deposit your laboratory protocols in protocols.io to enhance the reproducibility of your results. Protocols.io assigns your protocol its own identifier (DOI) so that it can be cited independently in the future. For instructions see: https://journals.plos.org/plosone/s/submission-guidelines#loc-laboratory-protocols. Additionally, PLOS ONE offers an option for publishing peer-reviewed Lab Protocol articles, which describe protocols hosted on protocols.io. Read more information on sharing protocols at https://plos.org/protocols?utm_medium=editorial-email&utm_source=authorletters&utm_campaign=protocols.

As the corresponding author, your ORCID iD is verified in the submission system and will appear in the published article. PLOS supports the use of ORCID, and we encourage all coauthors to register for an ORCID iD and use it as well. Please encourage your coauthors to verify their ORCID iD within the submission system before final acceptance, as unverified ORCID iDs will not appear in the published article. *Only*  the individual author can complete the verification step; PLOS staff *cannot*  verify ORCID iDs on behalf of authors.

We look forward to receiving your revised manuscript.

Kind regards,

Osman El-Maarri, Ph.D

Academic Editor

PLOS One

Journal Requirements:

1. If the reviewer comments include a recommendation to cite specific previously published works, please review and evaluate these publications to determine whether they are relevant and should be cited. There is no requirement to cite these works unless the editor has indicated otherwise.

2. Please review your reference list to ensure that it is complete and correct. If you have cited papers that have been retracted, please include the rationale for doing so in the manuscript text, or remove these references and replace them with relevant current references. Any changes to the reference list should be mentioned in the rebuttal letter that accompanies your revised manuscript. If you need to cite a retracted article, indicate the article’s retracted status in the References list and also include a citation and full reference for the retraction notice.

[Note: HTML markup is below. Please do not edit.]

Reviewers' comments:

Reviewer's Responses to Questions

**Comments to the Author**

1. If the authors have adequately addressed your comments raised in a previous round of review and you feel that this manuscript is now acceptable for publication, you may indicate that here to bypass the “Comments to the Author” section, enter your conflict of interest statement in the “Confidential to Editor” section, and submit your "Accept" recommendation.

Reviewer #1: (No Response)

Reviewer #2: All comments have been addressed

Reviewer #3: All comments have been addressed

Reviewer #4: All comments have been addressed

**********

2. Is the manuscript technically sound, and do the data support the conclusions?

The manuscript must describe a technically sound piece of scientific research with data that supports the conclusions. Experiments must have been conducted rigorously, with appropriate controls, replication, and sample sizes. The conclusions must be drawn appropriately based on the data presented.

Reviewer #1: Partly

Reviewer #2: Partly

Reviewer #3: Yes

Reviewer #4: Yes

**********

3. Has the statistical analysis been performed appropriately and rigorously?

Reviewer #1: No

Reviewer #2: I Don't Know

Reviewer #3: Yes

Reviewer #4: Yes

**********

4. Have the authors made all data underlying the findings in their manuscript fully available?

The PLOS Data policy requires authors to make all data underlying the findings described in their manuscript fully available without restriction, with rare exception (please refer to the Data Availability Statement in the manuscript PDF file). The data should be provided as part of the manuscript or its supporting information, or deposited to a public repository. For example, in addition to summary statistics, the data points behind means, medians and variance measures should be available. If there are restrictions on publicly sharing data—e.g. participant privacy or use of data from a third party—those must be specified.

Reviewer #1: Yes

Reviewer #2: Yes

Reviewer #3: Yes

Reviewer #4: Yes

**********

5. Is the manuscript presented in an intelligible fashion and written in standard English?

PLOS ONE does not copyedit accepted manuscripts, so the language in submitted articles must be clear, correct, and unambiguous. Any typographical or grammatical errors should be corrected at revision, so please note any specific errors here.

Reviewer #1: Yes

Reviewer #2: Yes

Reviewer #3: Yes

Reviewer #4: Yes

**********

6. Review Comments to the Author

Please use the space provided to explain your answers to the questions above. You may also include additional comments for the author, including concerns about dual publication, research ethics, or publication ethics. (Please upload your review as an attachment if it exceeds 20,000 characters)

Reviewer #1: In the revised manuscript, the authors have addressed some of the critiques raised, but several critical issues remain inadequately addressed.

Major points

1. Responding to the original comment “It is not clear what is the trigger of blood coagulation in the microfluidic device (the contact pathway is blocked with corn trypsin inhibitor, and no tissue factor is mentioned for this experimental setting)”, the authors state that “the trigger of blood coagulation is the mechanical injury causing disruption of the endothelial cell monolayer and exposure of the subendothelial coating of collagen”. It remains unclear how mechanical injury and collagen exposure can trigger clotting when the contact pathway is blocked, and no tissue factor is present in the experimental setup used in this study.

2. In response to the original critique regarding the sample size of the ROTEM measurements, the authors state that “despite the small sample size and missing statistics, we believe the results do show a clear trend and argue for an effect of TG2 on clot formation”. However, the lack of funds does not justify including these preliminary results, as their interpretation is not adequately supported and could be misleading. For example, referring to Fig. 3 and the numeric parameters reported in Table 1, the authors claim a dose-dependent effect of TG2 on MCF. However, if measurements from a larger sample show that one of the two values currently reported for 40 µg/mL TG2 (12 and 18) is an outlier, then the MCF at all 3 doses would be the same, and the current conclusion would be invalid. Therefore, the small-sample ROTEM data should be excluded from this report if sufficiently large samples cannot be measured.

3. Regarding the analysis of the turbidimetric curves, the authors state that “it is not essential to use complete lysis as baseline”. I agree with this view if the turbidity at the start and end of the process is the same. This generally holds for clot lysis of pure fibrin, but not always for more complex plasma clots. This issue is clearly illustrated by the curves in the supplementary Figure 5. How can the authors interpret values below 1 at the end of lysis if they use the difference between the start-point absorbance and the maximal absorbance to estimate 50% lysis? I also agree that the time from 50% clotting to 50% lysis is a reliable estimate of lytic efficiency. If the authors acknowledge this, they should replace all figures showing the time from Amax (which is rather arbitrary) to 50% lysis with those added to the revised supplement.

Reviewer #2: I find the revised manuscript to be significantly improved, and I thank the authors for taking the time to address my comments. I have only minor comments regarding data presentation which I believe should be considered prior to publication.

• For clarity, it would be helpful if details about the number of replicates were added to the figure legend in addition to the methods. For example, the legend for figure 1 could include “Experiments were performed with blood from 6 unique donors (n=6),” and the legend for the turbidity experiments could include “Experiments were performed in 5 unique replicates with the same batches of plasma (n=5). ”

• Unless they are just extremely difficult to see, several microscopy images still appear to be lacking scale bars (Fig. 1A, 1B, bottom two panels of 1D). In other images (e.g., Fig 1D top set of images), a scale bar is included, but it does not appear to be indicated anywhere what distance the scale bar corresponds to.

• In Figure 4 B-D, the phrase “dotted lines indicate direct comparisons between paired samples,” is somewhat misleading. Because these are two different sources of plasma, they cannot truly be “paired.” I fully agree, however, that this is an appropriate comparison for the other three groups (FXIII-deficient plasma with TG2 or FXIII). I believe it would be most appropriate to remove the dotted line between the black symbols and pink symbols. If the authors wish to include the reference plasma from the same experimental replicate for comparison, they could consider using different shapes for the symbols or something similar.

Reviewer #3: I thank the authors for their thorough responses to the previous review comments and for the substantial revisions made to the manuscript. The revised version provides a significantly strengthened and more balanced assessment of the potential role of transglutaminase 2 (TG2) in fibrin clot stabilization. The combination of a microfluidic endothelialized bleeding model, viscoelastic analyses, clot formation and lysis assays, and proteomic characterization provides a coherent body of evidence supporting the conclusion that TG2 is capable of contributing to fibrin crosslinking and clot stabilization. Importantly, the authors have appropriately acknowledged the limitations of the study, particularly regarding the physiological relevance of endogenous TG2 and the preliminary nature of the therapeutic implications. The conclusions are now generally well aligned with the presented data. The proteomic analyses revealing distinct crosslinking patterns and differential protein incorporation between TG2-mediated and FXIII-mediated clots add further value to the manuscript and provide a useful basis for future mechanistic studies. Only minor typographical and language issues remain and can be addressed during final editing. Overall, I believe the manuscript is technically sound, the conclusions are supported by the data presented, and the work will be of interest to researchers studying coagulation, fibrin clot biology, and transglutaminase function. I support publication in PLOS ONE.

Reviewer #4: The authors have addressed all comments and suggestions. I believe that the current state of the manuscript is sound with added information from the other reviewers and am happy for it to published in it's current state.

**********

7. PLOS authors have the option to publish the peer review history of their article (what does this mean?). If published, this will include your full peer review and any attached files.

If you choose “no”, your identity will remain anonymous but your review may still be made public.

**Do you want your identity to be public for this peer review?**  For information about this choice, including consent withdrawal, please see our Privacy Policy.

Reviewer #1: No

Reviewer #2: No

Reviewer #3: **Yes:**  Arijit Biswas

Reviewer #4: No

**********

[NOTE: If reviewer comments were submitted as an attachment file, they will be attached to this email and accessible via the submission site. Please log into your account, locate the manuscript record, and check for the action link "View Attachments". If this link does not appear, there are no attachment files.]

To ensure your figures meet our technical requirements, please review our figure guidelines: https://journals.plos.org/plosone/s/figures

You may also use PLOS’s free figure tool, NAAS, to help you prepare publication quality figures: https://journals.plos.org/plosone/s/figures#loc-tools-for-figure-preparation.

NAAS will assess whether your figures meet our technical requirements by comparing each figure against our figure specifications.

# Author comment: Author response to Decision Letter 2

**DOI:** 10.1371/journal.pone.0354080.r004

1 Jul 2026

Please refer to the separate document "Response to Reviewers".

Attachment Submitted filename: Response to Reviewers_Revision2.docx

# Editor report: Decision Letter 2

**DOI:** 10.1371/journal.pone.0354080.r005

**Authors:** Osman El-Maarri

2 Jul 2026

The role of transglutaminase 2 in stabilizing fibrin clots and supporting hemostasis

PONE-D-26-00575R2

Dear Dr. Schroeder,

We’re pleased to inform you that your manuscript has been judged scientifically suitable for publication and will be formally accepted for publication once it meets all outstanding technical requirements.

Within one week, you’ll receive an e-mail detailing the required amendments. When these have been addressed, you’ll receive a formal acceptance letter and your manuscript will be scheduled for publication.

An invoice will be generated when your article is formally accepted. Please note, if your institution has a publishing partnership with PLOS and your article meets the relevant criteria, all or part of your publication costs will be covered. Please make sure your user information is up-to-date by logging into Editorial Manager at Editorial Manager® and clicking the ‘Update My Information' link at the top of the page. For questions related to billing, please contact billing support.

If your institution or institutions have a press office, please notify them about your upcoming paper to help maximize its impact. If they’ll be preparing press materials, please inform our press team as soon as possible -- no later than 48 hours after receiving the formal acceptance. Your manuscript will remain under strict press embargo until 2 pm Eastern Time on the date of publication. For more information, please contact onepress@plos.org.

Kind regards,

Osman El-Maarri, Ph.D

Academic Editor

PLOS One

Additional Editor Comments (optional):

Reviewers' comments:

# Editor report: Acceptance letter

**DOI:** 10.1371/journal.pone.0354080.r006

**Authors:** Osman El-Maarri

PONE-D-26-00575R2

PLOS One

Dear Dr. Schroeder,

I'm pleased to inform you that your manuscript has been deemed suitable for publication in PLOS One. Congratulations! Your manuscript is now being handed over to our production team.

At this stage, our production department will prepare your paper for publication. This includes ensuring the following:

* All references, tables, and figures are properly cited

* All relevant supporting information is included in the manuscript submission,

* There are no issues that prevent the paper from being properly typeset

You will receive further instructions from the production team, including instructions on how to review your proof when it is ready. Please keep in mind that we are working through a large volume of accepted articles, so please give us a few days to review your paper and let you know the next and final steps.

Lastly, if your institution or institutions have a press office, please let them know about your upcoming paper now to help maximize its impact. If they'll be preparing press materials, please inform our press team within the next 48 hours. Your manuscript will remain under strict press embargo until 2 pm Eastern Time on the date of publication. For more information, please contact onepress@plos.org.

You will receive an invoice from PLOS for your publication fee after your manuscript has reached the completed accept phase. If you receive an email requesting payment before acceptance or for any other service, this may be a phishing scheme. Learn how to identify phishing emails and protect your accounts at https://explore.plos.org/phishing.

If we can help with anything else, please email us at customercare@plos.org.

Thank you for submitting your work to PLOS ONE and supporting open access.

Kind regards,

PLOS ONE Editorial Office Staff

on behalf of

Priv.-Doz. Dr. Osman El-Maarri

Academic Editor

PLOS One
